# Trust Buffers Against Reduced Life Satisfaction When Faced With Financial Crisis

**DOI:** 10.3389/fpsyg.2021.632585

**Published:** 2021-06-24

**Authors:** Jocelyne Clench-Aas, Ingrid Bergande, Ragnhild Bang Nes, Arne Holte

**Affiliations:** ^1^Division of Mental and Physical Health, Department of Public Health Science, Norwegian Institute of Public Health, Oslo, Norway; ^2^Faculty of Landscape and Society, Norwegian University of Life Sciences, Ås, Norway; ^3^Department of Psychology, Promenta Research Center, University of Oslo, Oslo, Norway; ^4^Department of Psychology, University of Oslo, Oslo, Norway

**Keywords:** well-being, income, trust, satisfaction, financial crisis, Europe

## Abstract

**Background:** In light of the coronavirus disease 2019 (COVID-19) pandemic and its large economic consequences, we used a three-layer nested structural model (individual, community, and country), each with a corresponding measure of income, trust, and satisfaction, to assess change in their interrelationships following a global crisis; which, in this study, is the 2008/2009 financial crisis.

**Methods:** With multilevel techniques, we analyzed data from two waves (2006 and 2012) of the European Social Survey (ESS) in 19 countries (weighted *N* = 73,636) grouped according to their levels of trust.

**Results:** In high trust countries, personal life satisfaction (LS) was not related to personal, community, or national income before or after the crisis. In contrast, in low trust countries, LS was strongly related to all three forms of income, especially after the crisis. In all country groups, personal, social, and political trust moderated their respective effects of income on LS (“the buffer hypothesis”). Political trust moderated the effects of income more strongly in low trust countries. The moderating effect of political trust increased sharply after the crisis. After the crisis, national-level factors (e.g., political trust, national income) increased their importance for LS more than the factors at the local and individual levels. However, the relative importance of all the three forms of income to LS increased after the crisis, to the detriment of trust.

**Conclusion:** Economic crises seem to influence personal LS less in high trust countries compared with low trust countries. Hence, high trust at a national level appears to buffer the negative impact of a financial crisis on personal satisfaction. Overall, the factors at the national level increased their impact during the financial crisis. When facing a global crisis, the actions taken by institutions at the country level may, thus, become even more important than those taken before the crisis.

## Introduction

Global crises are an integral part of societies, be it terrorist attacks, environmental crises, pandemics, or economic crises. The speed at which some of these crises become global seems to increase. At the same time, countries seem to vary considerably in how they tackle such crises and how these crises affect their population. Such rapid increase in dispersion and severity of crises challenge both national and international authorities in finding effective, efficient, and fair measures to take.

Major global events, such as the coronavirus disease 2019 (COVID-19) pandemic and the 2008/2009 financial crisis, may have large repercussions on individuals, the social fabric of the society, and, as a result of changes in the economy of a nation, the way a country is run (Geys and Qari, 2017; Castells et al., 2018). In turn, this may influence satisfaction with how the country is run, and thus indirectly influence subjective well-being (SWB) and trust (Helliwell et al., 2018).

Subjective well-being is of obvious importance for people, personally, emotionally, and cognitively. During times of crisis, the levels of SWB are at risk, directly and indirectly, for example, through changing labor market opportunities or changing trust levels (WHO, 2011). Furthermore, SWB is also influenced by income, both within and between countries.

This has been discussed for a long time, and by authors previously (Clench-Aas and Holte, 2017). In 1974, Easterlin claimed that “at a point in time happiness varies directly with income, but over time happiness does not increase when a country's income increases.” He also hypothesized that together with increasing income, the aspirations of people increase. When aspirations fail to be met, well-being is diminished.

Deaton (2008) contested this claim and rather suggested that the well-known relationship between life satisfaction (LS) and Ln gross domestic product (ln GDP) is linear with no upper satiation point. Easterlin countered that these changes represent short-term changes in GDP. Long-term changes, for example, over 30 years, show no change, regardless of the type of country: developed, developing, or in transition (Easterlin, 1974, 1995; Deaton, 2008; Stevenson and Wolfers, 2008; Easterlin and Angelescu, 2009).

Most of the literature was associated with the effect of increased income on well-being. Less has been studied concerning the effect of decrease in income and well-being. Yet, there are indications that the effect of decreased income is much stronger than a comparable increase in income and can have important mental health consequences (Novemsky and Kahneman, 2005; Boyce et al., 2013).

Trust, be it social or national, is an integral part of the social capital inherent in a country. This concerns personal trust in terms of self-confidence and self-esteem, social trust in terms of trusting other people, and political trust in terms of having trust in how the country is run.

Most people would prefer to feel well and live in a high-trust society rather than in a low-trust one. However, SWB and trust are also politically important as they facilitate the economy by being associated with high work productivity, effectiveness, and creativity, as well as good health and social relationships between people (Fredrikson, 2001; Diener and Seligman, 2004; Lyubomirsky et al., 2005a,b; Andreasson, 2017; Diener et al., 2017).

Trust and well-being play an important role in rebuilding society after a global crisis. Therefore, it is paramount to understand how crises impact well-being and trust in populations, and what measures are needed to strengthen the well-being and trust of individuals.

The financial crisis of 2008/2009 was a major world event. Many countries also experienced a second recession around 2011. The rise in unemployment and suicide rates, as well as the general decrease in public health that resulted from the crisis, created important challenges for the political leaders and for the individuals themselves (Stuckler et al., 2011; Chang et al., 2013; Karanikolos et al., 2013; Toffolutti and Suhrcke, 2014).

However, the impact of the crisis differed between countries and regions because of factors, such as the economic situation before the crisis, availability of public safety nets, and how political and economic institutions initially responded to the crisis (Arampatzi et al., 2019). Some countries employed austerity policies, whereas others increased funding for health services and used other fiscal stimuli to minimize the influence of the crisis (Stuckler et al., 2009, 2011; Karanikolos et al., 2013; Stuckler and Basu, 2013).

In Europe, large population groups experienced unemployment, loss of homes, decreased income, loss of benefits such as pensions, and several other major life events (Heretick, 2013). Initially, such exposures tend to be characterized by a strong fall in LS, followed by a gradual recovery (Luhmann et al., 2012). Less attention has been given to factors that might mitigate the negative effects. Yet, there is no doubt that the sudden fall in income experienced by some was detrimental to their SWB (Novemsky and Kahneman, 2005; Boyce et al., 2013) and their sense of self-identity and social identity (Heretick, 2013).

The mechanisms behind these changes are still unknown. However, following the theory of social identity proposed by Tajfel (1978), the impact may occur through several channels or layers. As pointed out by Tajfel (1978), individuals are surrounded by family and friends, neighbors, colleagues and also have an identity connected to the country and the environment they live in. The original sentence must be kept Accordingly, the individual's concept of social identity to the groups in which the individual belongs, is closely related to the individual's physical and mental health and well-being (Stets and Burke, 2000; Abrams and Hogg, 2006; Jetten et al., 2017). For short periods after the financial crisis, lower levels of personal LS have been reported in the USA and Europe (Deaton, 2008; Clench-Aas and Holte, 2017). In Iceland, 2007–2009, however, the crisis had only a limited association with personal LS (Gudmundsdottir, 2013). There is also evidence that the financial crisis had a negative effect on trust in many countries (Stevenson and Wolfers, 2011; Habibov and Afandi, 2015; Navarro-Carrillo et al., 2018; Ananyev and Guriev, 2019; Daskalopoulou, 2019). Some studies have reported that higher levels of social capital and trust provide resilience that allows some countries to handle economic crises better than others (Helliwell et al., 2014a, 2016).

Resilience to decreased LS in some countries could be explained through a moderation by quality of governance, most likely through generating trust (Arampatzi et al., 2019). This was seen especially in transition countries where declines in political satisfaction and personal LS were associated with declines in political trust and the reduced association between social trust and well-being after the crisis (Habibov and Afandi, 2015). However, this was most prominent 2–5 years after the crisis (Bartolini et al., 2017).

The regions of Europe did not react equivocally to the financial crisis. This was seen and often attributed to differences in social capital (Rodríguez-Pose, 2012; Bjørnskov, 2014). The northern region, primarily Nordic countries, and the transition countries are especially signalized (Rodríguez-Pose, 2012; Bartolini et al., 2017).

However, no one has yet investigated holistically and simultaneously the relative importance of different socio-structural layers of society, such as the individual, local social community, and country; or the different roles of trust in these socio-structural layers; or how the relationships between income, trust, and satisfaction are affected by a major global crisis.

Such knowledge may be crucial in finding the best options to maintain or increase satisfaction with life and social and political satisfaction when confronted with major global crises. Part of the uncertainty of how and to what degree environmental factors may influence societies in general and in times of crisis may be related to not accounting for the relative importance of factors in different socio-structural layers of society (Schyns, 2002; Clench-Aas and Holte, 2018).

In a previous study (Clench-Aas and Holte, 2021), we have discussed the holistic multilevel model of Dahlgren and Whitehead (1991) of social determinants of health, the “rainbow model,” which was built on Bronfenbrenner (2009). The holistic model aims to conceptualize how economic, environmental, and social inequalities may determine the risk of people getting ill, their ability to prevent sickness, or their access to effective treatments. The model placed individuals at the center of the model, with its fixed factors, such as sex, age, and constitutional endowment. Surrounding them were different layers of modifiable health determinants, such as individual lifestyles, social and community networks, and economic, cultural, and physical environments.

This framework has inspired researchers to construct a range of hypotheses about the determinants of health and explore their relative influence on different health outcomes. Lately, the model has also been expanded to include mental health (Clench-Aas and Holte, 2021).

To achieve a comprehensive understanding of how the financial crisis of 2008/2009 possibly changed the relationship between income and satisfaction, we have, inspired by Dahlgren and Whitehead, launched a similar theoretical model where we regard society as a construction with several socio-structural layers (Clench-Aas and Holte, 2021). Much like the conceptualization of social identity by Tajfel (1978), we assumed that several layers can influence the well-being of an individual.

The main determinants of well-being are layered from the individual to the national structural layer. Each layer may influence the well-being of the individual. We define the individual as the basic unit (micro layer). We then regard the individual as nested into her or his local community (mezzo layer), which, again, is nested into the country (macro layer).

Likewise, we regard the economy of the individual as nested into the economy of his/her local community, which, again, is nested into the national economy. Correspondingly, we regard how satisfied individuals are with their life, as nested into how satisfied individuals are with their local community, which again is nested into how satisfied individuals are with how their country is run.

We, then, hypothesize that there is an association between income, trust, and satisfaction within each layer. In addition, we hypothesize that trust, i.e., personal trust, social trust, and political trust, modifies the associations between income and satisfaction within each layer. In particular, we hypothesize that the personal, social, and political forms of trust act as buffers against the effect of personal income on personal LS (“the buffer hypothesis”).

Understanding how all these parameters relate to each other may provide a deeper and more holistic comprehension of how societies work and how global crises impact these mechanisms. Until we have analyzed these associations together in one and the same model, taking into account the effects from all three layers of society, there may be difficulties in interpreting the consequences of the unique relationships. These are the basic concepts used in the model:

Personal income, also called absolute income, refers to the annual personal income of an individual, e.g., household income. There is good evidence that personal income influences LS/well-being (Biswas-Diener, 2008; Diener et al., 2018). Unfortunately, income does not account for the expenses or debt that families have.

Community income refers to the generalized income of the population of the local community, that is, either a poor or rich community. However, the concept of an average community income at the local level is rather complex. It may reflect the generalized level of wealth seen, for example, finer stores, homes, cars, etc., in richer communities, or more government-oriented facets, such as crime and social and mental health problems in poorer communities (Brodeur and Flèche, 2018).

Many studies have found negative associations between well-being and community income at the highest geographical level, such as district, province or county, state, or metropolitan statistical area (Blanchflower and Oswald, 2004; Kingdon and Knight, 2007; Barrington-Leigh and Helliwell, 2008; Graf and Tillé, 2013; Brodeur and Flèche, 2018). However, at the lowest or neighborhood level, most observed are positive associations between well-being and community income (Kingdon and Knight, 2007; Barrington-Leigh and Helliwell, 2008; Knies et al., 2008; Clark et al., 2009; Dittmann and Goebel, 2010; Brodeur and Flèche, 2018; Ma et al., 2018).

National income refers to the total income of a country, e.g., in terms of GDP. Although evidence and opinions are quite contradictive, some evidence indicates that GDP influences LS/well-being (Biswas-Diener, 2008; Diener et al., 2018), especially in the transition countries (Easterlin, 2009; Bartolini et al., 2017). There is a general agreement that income is an important factor for LS. However, a disagreement exists about the extent to which income influences personal LS and whether national, relative, or absolute income matters the most (Easterlin, 1995; Biswas-Diener, 2008; Caporale et al., 2009; Diener et al., 2013, 2018).

Personal trust refers to the trust of an individual in her- or him-self, e.g., self-confidence or self-esteem. High self-esteem and happiness are closely related (Trzesniewski et al., 2006; Orth et al., 2012; Kuster et al., 2013; von Soest et al., 2016). The notion of self-trust was thoroughly described by Govier (1993) and is integrated into the notion of social trust while maintaining an independent dimension.

Longitudinal studies indicate that the relationship is in the direction of self-esteem to happiness (Baumeister et al., 2003; Lyubomirsky et al., 2006; Margolis and Lyubomirsky, 2018). Researchers have debated whether personal trust has any influence on important life outcomes (Baumeister et al., 2003; Swann et al., 2007, 2008; Krueger et al., 2008), yet several studies suggest that strengthened individual self-esteem increases well-being through improved social relationships (Johnson and Galambos, 2014; Marshall et al., 2014; Orth et al., 2014; Mund et al., 2015), mental health (Orth et al., 2008, 2016; Sowislo and Orth, 2013; Wouters et al., 2013; Sowislo et al., 2014; Steiger et al., 2014), school and education (Trzesniewski et al., 2006; von Soest et al., 2016), work (Trzesniewski et al., 2006; Orth et al., 2012; Kuster et al., 2013; von Soest et al., 2016), and physical health (Trzesniewski et al., 2006; Orth et al., 2012; Orth and Robins, 2013). For reviews, see the following references (Donnellan et al., 2011; Orth and Robins, 2013, 2014; Orth, 2018). Altogether, according to Orth and Robins (2019), these studies allow for relatively strong conclusions because many of the studies used large and representative samples, controlled for prior levels of the outcomes, and controlled for confounding factors such as gender, socioeconomic status, intelligence, and life events.

Social trust, also called interpersonal trust, refers to having trust in other people. The effect of trust on well-being has been shown to exceed that of GDP over the long term. The effect of GDP, which was prominent over the short term, was considerably reduced in favor of social trust over the long term (Bartolini and Sarracino, 2014). When examining national differences in happiness, social trust has been shown to constitute a powerful explanatory factor (Bjørnskov, 2003; Helliwell, 2003; Rodríguez-Pose, 2012; Helliwell et al., 2016; Glatz and Eder, 2020).

Political trust refers to having trust in the national institutions, in particular the political institutions (Khodyakov, 2007). Political trust encompasses the degree to which a person trusts the given institution to fulfill its role.

The actual performance of the institution seems to influence the evaluation of political trust (Hudson, 2006; Helliwell et al., 2014b, 2016, 2018). Political trust seems to be associated with both personal LS (Mota and Pereira, 2008) and happiness (Hudson, 2006). One study found political trust and political satisfaction to be stronger predictors of personal LS than trust in the social environment (Mota and Pereira, 2008). These findings were not confirmed in the USA (Bartolini et al., 2013b), and were considered spurious and reflective of other factors in Europe (Glatz and Eder, 2020).

There is a general agreement that trust is related to well-being (Helliwell and Huang, 2008; Helliwell and Wang, 2011; Helliwell et al., 2016).

Personal LS commonly refers to an individual's long-term, cognitive evaluation of one's life as a whole, as opposed to happiness, which commonly refers to a more emotional or affective experience of joy or well-being (Eid and Diener, 2004). Both personal LS and happiness are subjective measures and are commonly used to indicate well-being (Veenhoven, 1996; Dolan et al., 2008).

Social satisfaction refers to the propensity of an individual to be satisfied with her or his local social environment, e.g., closeness, respectfulness, safety, and helpfulness (Bárcena-Martín et al., 2017). Whereas one's personal economy contributes to community satisfaction (Fitz et al., 2016), less is known about how community income contributes to social satisfaction. However, social and community satisfaction and social interaction are known to contribute positively to individual well-being (Theodori, 2001; Bárcena-Martín et al., 2017).

Political satisfaction refers to an individual's satisfaction with how one's country is run, e.g., economy, democracy, education, health services, police, politicians, government, and national leadership.

There is good evidence that political satisfaction influences personal well-being (Helliwell et al., 2018; Clench-Aas and Holte, 2021).

Good policy development in terms of facilitating for a population who is satisfied with life, their social environment, and how the country is run, may depend on which of these parameters have the greatest positive effects on their well-being. Therefore, we examined the relationships between income, satisfaction, and trust in three layers, namely, personal, community, and national, in 19 European countries before and after the major financial crisis in 2008/2009.

The study has the following four aims:

- To assess how the relationship between income and satisfaction within different layers of the society when accounting for personal, social, and political trust was changed from before to after the financial crisis of 2008/2009.
- To assess if countries, grouped according to their levels of trust, differ in the importance of the financial crisis on LS.
- Holistically, to determine the relative importance of the financial crisis to individual LS, after accounting for all variables of income and trust in each layer, i.e., individual, community, and country.
- To determine if the eventual buffering role of trust on the relationship between income and satisfaction within each layer holistically changed after exposure to the financial crisis (“The buffer hypothesis”).

## Materials and Methods

We used data from the European Social Survey (ESS), which has developed standards regarding sample selection, translation of the questionnaire, data collection and processing, and documentation to ensure that the same methodology is used in all participating countries. This ensures that the data are representative and highly comparable across nations. There has been a high response rate in all rounds, with a mean of 65.8% in the last assessment round. The sample consists of individuals aged 15 and over and sampling is conducted through strict random probability methods. The questionnaire is made up of a core module and two rotating modules. The data were collected through face-to-face interviews lasting approximately for 1 h.

In this study, the data were restricted to the years completed with respect to the choice of variables. Thus, we used the cumulative dataset for rounds 3 and 6 (corresponding to 2006 and 2012), found on the ESS web page (www.europeansocialsurvey.org). Data from the respondents in the 19 countries who participated in both rounds, which include the variables of interest, were used. The final sample included 72,461 individuals (W–N = 73,307) with a mean age of 48 years and 54% were females (in the weighted sample 46 years and 51% females). Only data from the core ESS module were used in this study. Response rates for each year and country and the number of missing values for the different countries and parameters are presented in Supplementary Materials Tables - Tables 1 and 2, respectively (missing data were removed list-wise). Year represented investigation year.

### Measures

We specified a structural model with three nested layers: individual, community, and country (Clench-Aas and Holte, 2021). Each layer includes one measure of income, one on trust, and one on satisfaction. The three measures of income were personal income, community income, and national income. The three measures of trust were personal trust, social trust, and political trust. The three measures of satisfaction were LS, social satisfaction, and political satisfaction. The trust measures were used as moderator variables.

### Layer Defining Measures

We used three levels of analysis: (1) individual (micro), (2) local community (mezzo), and (3) country (macro). Refer to Methods in Supplementary Material 1 for more details in definitions of variables and an earlier article (Clench-Aas and Holte, 2021).

The micro layer was defined by the ID number of an individual.

The mezzo layer was defined by two nested variables: (a) the regions within each country and (b) social class. The regions within each country, as described on the ESS website, were defined as the nomenclature of territorial units for statistics, abbreviated as NUTS. Since the regions are in some cases rather large, and people tend to live in areas of rather similar social class, we defined the community layer as being both regions and social class.

The social class of the respondents was determined using education and occupation. We used a mean value for the respondent and his/her partner if present. If data on occupation or education was missing for the partner, we used the education and occupation of respondent. More detailed information is provided in Methods in Supplementary Material 1.

Macro layer was defined by 19 countries: Belgium (BE), Bulgaria (BG), Cyprus (CY), Denmark (DK), Finland (FI), France (FR), Germany (DE), Ireland (IE), The Netherlands (NL), Norway (NO), Poland (PL), Portugal (PT), Russia (RU), Spain (ES), Slovakia (SK), Slovenia (SI), Sweden (SE), Switzerland (CH), and the United Kingdom (GB).

### Income Measures

In the micro layer, personal income was measured in terms of the annual household income of the individual, based on the total net income of the household from all sources, that is, after-tax, national insurance, contributory pension payments, and so on. The income included not only earnings but state benefits, occupational, and other pensions, and unearned income such as interest from savings, rent, etc. More details concerning the calculation and methods used for standardizing the two measures of personal income, since the variable varied between the 2 years, are provided in Methods in Supplementary Material 1 and Supplementary Material 2 - Table 3.

In the mezzo layer, we used community income. Community income was calculated for this study as the aggregate of the household income value by country, region, and social class.

In the macro layer, national income was measured in terms of GDP, i.e., the sum of gross value added by all resident producers in the economy plus any product taxes minus any subsidies not included in the value of the products. The unit of measure was GDP per capita, PPP (current international $). For the analyses in this study, we used the log of GDP (Ln GDP) per capita divided by 1,000. For more details, refer to Methods in Supplementary Material 1.

### Trust Measures

Three trust variables were used as moderators. These were developed for the ESS, and have been in use since 2006 (Huppert et al., 2009).

In the micro layer, personal trust was measured in terms of self-esteem by the following item: “In general I feel very positive about myself.” Responses were given on a five-point scale ranging from 1 “Agree strongly” to 5 “Disagree strongly” (Huppert and So, 2013). The variable was recoded inversely (1–5) to comply with the scales used in the other questions.

In the mezzo layer, social trust was measured by the following item “Generally speaking, would you say that most people can be trusted, or that you can't be too careful in dealing with people?” Responses were given on an 11-point scale ranging from 0 to 10 (0 being “You can't be too careful” and 10 indicates “Most people can be trusted)” (Huppert et al., 2009; Helliwell et al., 2017). This measure of trust has been observed to be stable and its validity is confirmed (Bergh and Bjørnskov, 2014).

In the macro layer, political trust was measured by the five following items: “How much do you personally trust the country's parliament?”; “How much do you personally trust the police?”; “How much do you personally trust the legal system?”; “How much do you personally trust the politicians?”; and “How much do you personally trust the political parties?”. Responses to each were given on an 11-point scale ranging from 0 to 10 (0 indicates “you do not trust an institution at all” and 10 indicates “you have complete trust”) (Huppert et al., 2009; Helliwell et al., 2017). The answers were summed, yielding a parameter with a range of 0 to 50.

### Satisfaction Measures

#### Micro Layer

In the individual layer, personal LS was assessed by the following item “All things considered, how satisfied are you with your life as a whole nowadays?” Responses were given on an 11-point scale ranging from 0 to 10, 0 = “extremely dissatisfied” and 10 = “extremely satisfied.” This one-item scale is one of the most commonly used scales for assessing overall personal LS and shows moderate to high validity and reliability (Pavot et al., 1991).

#### Mezzo Layer

Social satisfaction was measured by a variable constructed as the average of the responses to four questions: (1) “Do you feel close to the people in local area?”, with response alternatives ranging from 1 = “disagree strongly” to 5 = “agree strongly;” (2) “Do you feel people treat you with respect?”; (3) “Do you feel people in local area help one another?”, both of the last questions ranging in response from 0 = “Not at all” to 6 = “A great deal;” (4) “Do you feel safe walking alone in local area after dark,” with response alternatives ranging from 1 = “Very unsafe” to 4 = “Very safe” (Cronbach's alpha = 0.593). These questions cover the areas of belonging, social support, respect, and safety in the local area. Questions 1 and 4 were extended to conform to the range of questions 2 and 3 (Nes et al., 2006). The final variable represented the average of the four questions.

#### Macro Layer

Political satisfaction was measured by a variable constructed as the sum of the responses to five questions, and that ranged in value from 0 to 50: (1) “How satisfied are you with the present state of the economy in your country?”; (2) “How satisfied are you with the national government?”; (3) How satisfied are you with the way democracy works in your country?”; (4) “How satisfied are you with the state of education in the country nowadays?”; and (5) “How satisfied are you with the state of health services in the country nowadays?”, all with responses given on an 11-point scale ranging from 0 to 10, 0 = “Extremely dissatisfied” and 10 = “Extremely satisfied.”

### Stratification of Countries According to Levels of Trust

Each of the 19 countries was ranked according to its level of social and political trust separately. The resulting rankings were added together, and a new ranking was performed of the combined value. The countries were then divided into three groups, Group 1, exhibiting the highest trust levels, included the Nordic countries of Denmark, Finland, Norway, and Sweden in addition to Switzerland and the Netherlands; Group 2, exhibiting a medium-trust level, included the United Kingdom, Belgium, Germany, Ireland, France, and Spain; and, finally, Group 3, exhibiting the lowest trust levels, included Slovenia, Cyprus, Slovakia, Russia, Portugal, Poland, and Bulgaria (see Table 1).

**Table 1:** Mean weighted estimates of social and political forms of trust are used in ranking and grouping countries into three groups.

**Trust group**	**Country**	**Social trust**	**Rank social trust**	**Political trust**	**Rank political trust**	**Combined rank/2**
High	Denmark	20.5	1	32.7	1	1
	Norway	19.8	2	29.6	3	2.5
	Finland	19.3	3	30.9	2	2.5
	Sweden	18.7	4	27.9	5	4.5
	Switzerland	17.8	5	29.2	4	4.5
	Netherlands	17.6	6	27.5	6	6
Medium	United Kingdom	16.9	7	22.7	9	8
	Germany	15.7	9	23.7	8	8.5
	Belgium	15.4	10	24.6	7	8.5
	Ireland	16.8	8	22.3	10	9
	Spain	15.1	11	19.8	13	12
	France	14.9	12	21.4	12	12
Low	Slovenia	14.0	13	18.1	14	13.5
	Cyprus	12.1	18	21.8	11	14.5
	Slovakia	12.8	15	18.1	15	15
	Russia	13.3	14	16.0	17	15.5
	Portugal	12.5	17	16.6	16	16.5
	Poland	12.6	16	15.9	18	17
	Bulgaria	10.8	19	11.4	19	19

### Confounders

The demographic variables adjusted for in all the analyses were a year of investigation, gender, age and age^2^, number of people living regularly as members of a household, marital status, mental health, being permanently sick or disabled, being unemployed, educational level, and occupational level. Being permanently sick or disabled and being unemployed were two alternatives in a question concerning main activity over the last 7 days. Mental health was a combination of two questions concerning feeling depressed or anxious. The two variables were recoded to either being most of the time or all the time depressed or anxious, as opposed to less than that. The two variables were then combined so that the individual had at least one of the two conditions. Age is well-documented to have a curvilinear relationship; and, therefore, it is highly recommended to use the squared function (Dolan et al., 2008).

### Statistical Analysis

The analyses were conducted using the Statistical Package of the Social Sciences (SPSS), version 25.0. All data were weighted in accordance with the ESS guidelines before conducting the analyses (Ganninger, 2007).

The primary method of analysis was multilevel analysis using the Linear Mixed models module in SPSS (Field, 2013). The data were weighted in these analyses using the post-stratification weight that includes a design weight. A three-level approach was used as the main method of analysis. The levels chosen were the individual; community, which, for practical purposes, was defined using two levels, (a) regions within each country and (b) social class (refer to section on measures, level defining measures); and country. To represent different hierarchical levels, a separate economic indicator was used for each layer; personal, community, and national income. The corresponding trust variables were personal trust, social trust, and political trust. The corresponding outcome variables were LS, social satisfaction, and political satisfaction. For each of these layers, gender, age and age^2^, the number of people in a household, marital status, education, occupation, being permanently sick or disabled, being unemployed, and mental health were entered as covariates.

**Figure 1:**
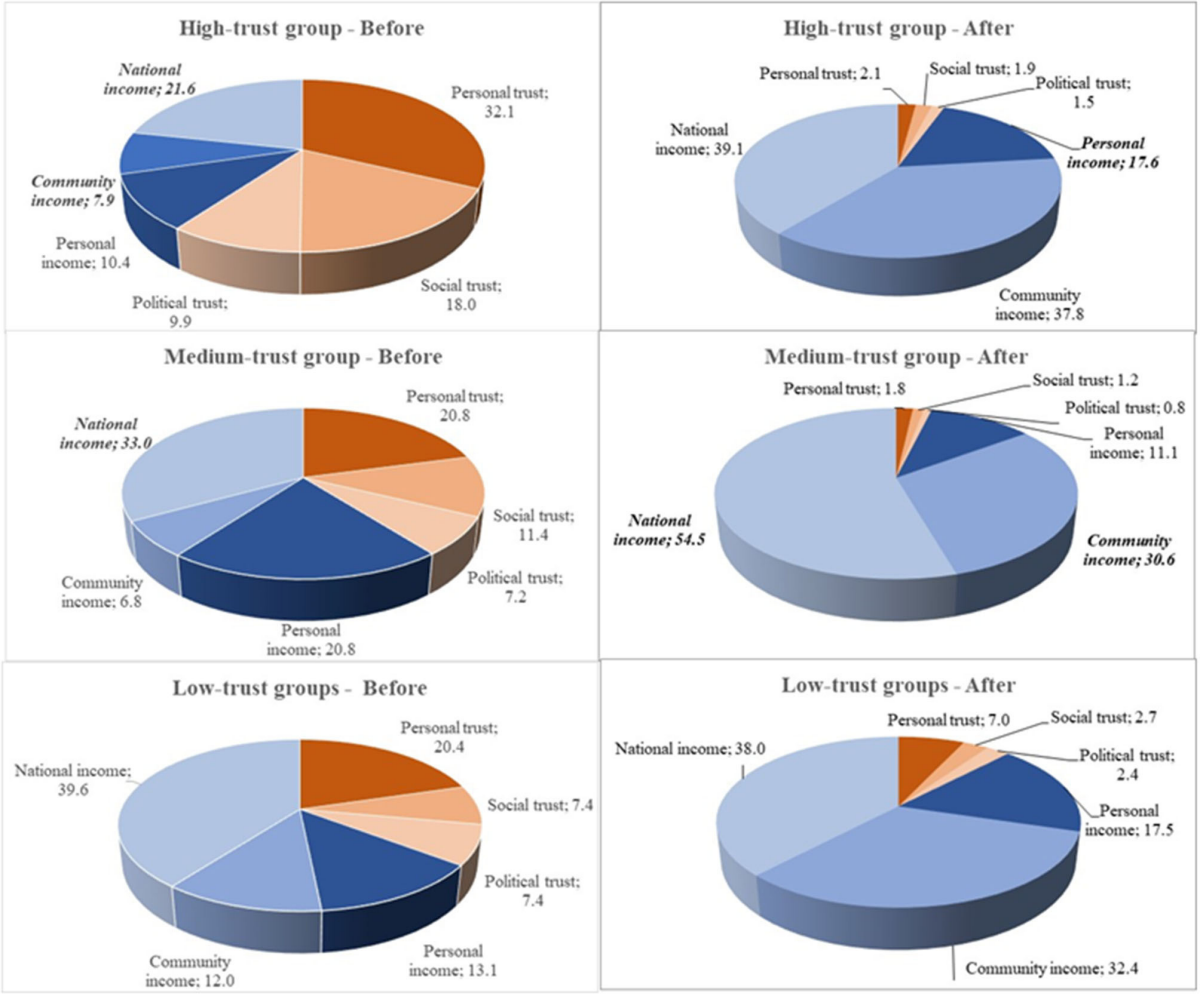
Overview of the relative effect of personal, relative, and national income, and personal, social, and national trust on LS. Results of multilevel analysis. Levels: individual, community and country. Separated by groups of countries stratified by overall trust and as before and after financial crisis 2008/2009. *N* = 73,307. Significant results are in bold and those that are negative are in italics.

**Figure 2:**
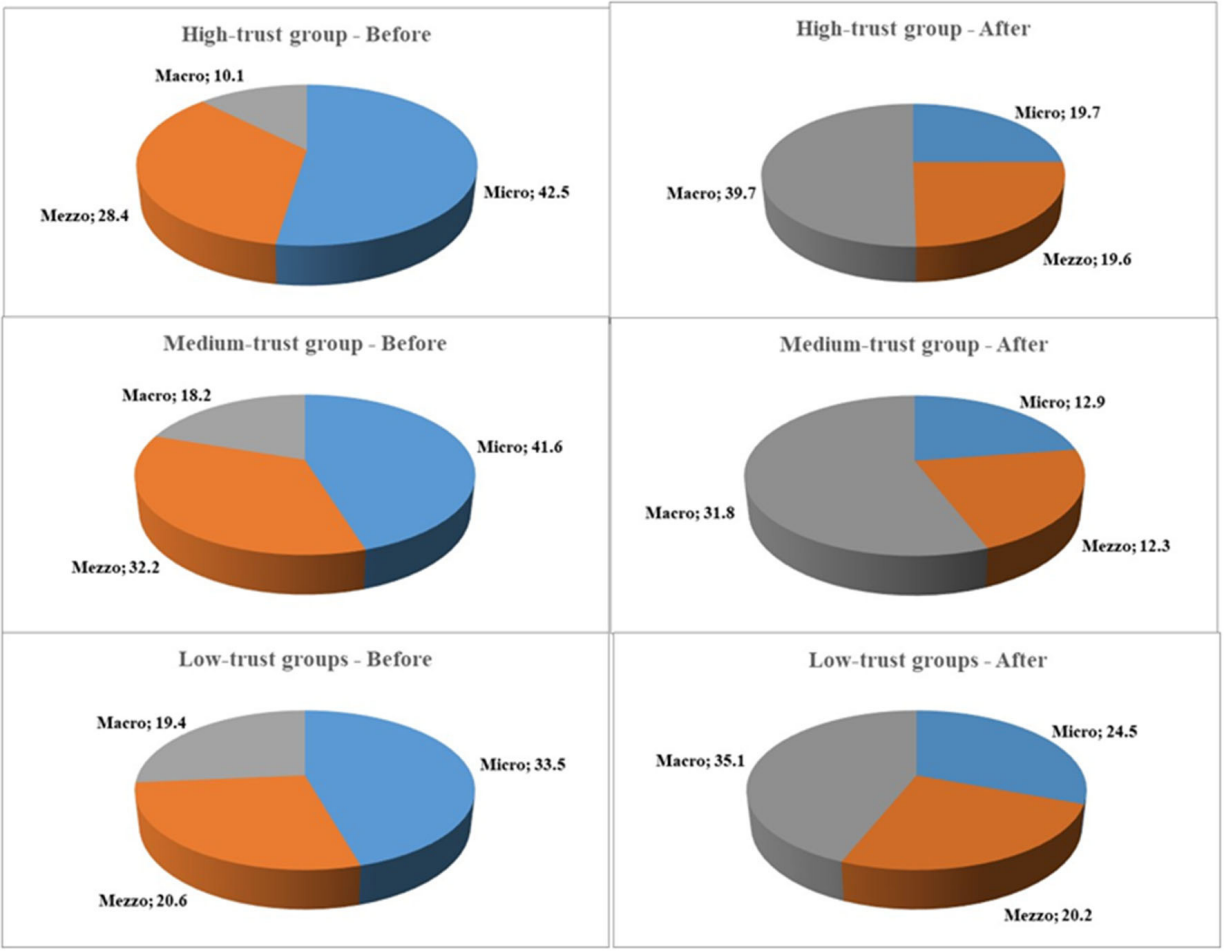
Overview of the relative effect of the three layers, micro, mezzo, and macro, on LS. Results of multilevel analysis. Levels: individual, community and country. Separated by groups of countries stratified by overall trust and as before and after financial crisis 2008/2009. *N* = 73,307.

For one series of analyses (**Table 4**), personal LS was the dependent variable with personal income, community income, national income, personal trust, social trust, and political trust as independent variables. This model was used in providing the coefficients used in Figures 1, 2. The same model was repeated for each of the stratified country groups defined by levels of trust.

The effect estimates calculated for Figures 1, 2 reflect the relative importance of a fixed set of parameters. For each trust group and for before and after a crisis, the relative importance of each parameter is calculated as follows:

(1) $$\begin{array}{r} \beta X1^{*}meanX1/\;\sum_{k=1}^{n=6}{(}_{k}^{n})\beta X^{*}meanX1\; \end{array}$$

For parameter k, n = number of parameters.

This method does not account for changes in variance or intercept. The intercepts were classified as random. Validation of using the different levels in multilevel analysis (entering country first, then community and social class as levels) was tested using the chi^2^ test based on differences in the Akaike Information Criterion (AIC) (log-likelihood) before and after entering levels. The estimation method was Restricted Maximum Likelihood. In the multilevel linear analyses, pseudo R^2^ was calculated by comparing the variances.

Moderation analyses were performed both by introducing an interaction in the multilevel analyses and by using Andrew F. Hayes' PROCESS tool for SPSS. The latter was, unfortunately, unable to incorporate multilevel analyses. However, the analyses were performed on the country groups based on overall trust where variation in trust was less than in the entire sample of 19 countries. The analyses were controlled for gender, age and age^2^, the number of people in a household, being sick or unemployed, and mental health.

Model fit was evaluated by significant R^2^ in the multiple linear regression. The assumption regarding multi-collinearity among the independent variables was not violated (all VIF values <3 and tolerance levels >0.2). The results were regarded as statistically significant at the 0.05 level. Unstandardized beta-coefficients with standard errors are also reported.

### Ethics

The data are available without restrictions for not-for-profit purposes.

In accordance with the ESS ERIC Statutes (Article 23.3), the ESS ERIC subscribes to the Declaration on Professional Ethics of the International Statistical Institute. The Research Ethics Committee reviews applications for studies for which the ESS ERIC is directly responsible, that is, which it directly contracts.

## Results

### Overall Description of the Country Groups

Table 1 shows the 19 countries divided into three groups according to a combination of levels of social and political trust.

### Before to After Crisis Between Country Groups

Table 2 shows that there were sizable differences in the primary parameters considered, between the groups of countries, and between before and across the crisis. The high trust group scored highest and the low group lowest on all variables of income, trust, and satisfaction, except for the level of personal trust between the high and low trust group.

**Table 2:** Weighted means of the primary variables of interest in the study before or after the financial crisis, with % change and significance of change by country group according to trust level.

**Country group**		**Before crisis**		**After crisis**		**% Change**
		**Mean**	**SE**	**Mean**	**SE**	
High	Personal income	46.71	0.34	53.95	0.35	15.5^*^
	Community income	46.30	0.20	53.65	0.24	15.9^*^
	National income	3.72	0.00	3.89	0.00	4.6^*^
	Personal trust	3.85	0.01	3.93	0.01	2.1^*^
	Social trust	18.84	0.04	18.91	0.04	0.4
	Political trust	29.06	0.08	30.16	0.08	3.8^*^
	Life satisfaction	7.93	0.02	8.12	0.02	2.4^*^
	Social satisfaction	4.62	0.01	4.74	0.01	2.6^*^
	Political satisfaction	6.34	0.01	6.53	0.01	3.0^*^
Medium	Personal income	38.05	0.32	27.43	0.20	−27.9^*^
	Community income	37.21	0.17	26.91	0.13	−27.7^*^
	National income	3.56	0.00	3.71	0.00	4.2^*^
	Personal trust	3.81	0.01	3.89	0.01	2.1^*^
	Social trust	15.73	0.04	16.01	0.04	1.8^*^
	Political trust	22.83	0.08	22.52	0.08	−1.4^*^
	Life satisfaction	7.08	0.02	7.15	0.02	1.0^*^
	Social satisfaction	4.32	0.01	4.56	0.01	5.6^*^
	Political satisfaction	5.11	0.02	4.95	0.02	−3.1^*^
Low	Personal income	11.75	0.16	10.86	0.10	−7.6^*^
	Community income	12.22	0.10	11.15	0.06	−8.8^*^
	National income	2.94	0.00	3.21	0.00	9.2^*^
	Personal trust	3.89	0.01	3.95	0.01	1.5^*^
	Social trust	12.70	0.06	12.63	0.05	−0.6
	Political trust	17.83	0.10	15.18	0.09	−14.9^*^
	Life satisfaction	5.99	0.02	6.13	0.02	2.3^*^
	Social satisfaction	4.33	0.01	4.40	0.01	1.6^*^
	Political satisfaction	4.25	0.02	3.77	0.02	−11.3^*^

*Weighted N = 73,307.*

* *Significantly higher at the 0.05% level, using t-test (testing differences between before and after the crisis)*.

Table 2 also shows that in the high trust group, all parameters except social trust increased significantly from before to after the crisis. However, in the other groups, the results were more mixed.

Personal and community income increased substantially in the high trust group, decreased substantially in the medium trust group, and decreased importantly but less in the low trust group. However, change in personal income should be interpreted with some caution since slightly different methods were used in the two survey years. National income increased in all three groups, but especially in the low trust group. In the high trust group, the change in national income was rather small.

Personal trust increased slightly in all groups. Changes in social trust were slight and not significant in the high and low groups but increased slightly in the medium group. Political trust increased in the high trust group and decreased in the medium and, especially, in the low trust group.

Personal LS and social satisfaction increased slightly in all groups, whereas political satisfaction declined in the medium trust and especially the low trust countries.

The differences from before to after the crisis in the variables varied considerably between single countries. For detailed results refer to Supplementary Material 2 - Table 5. Additional information on the correlation between the variables before and after the crisis can be found in Supplementary Material 2 - Table 4.

### Results by Layers

Table 3 presents the results of analyses layer by layer for each country group from before to after the crisis.

**Table 3:** Results [fixed effects (Beta (SE) sig)] and pseudo R^2^ for LS, social satisfaction, and political satisfaction as a function of their personal, community, and national income and trust parameters, respectively, before and after the financial crisis of 2008/2009.

	**Layer**	**Micro**				**Mezzo**				**Macro**			
**Country group**	**Measure of satisfaction mean/SE/Sig**	**LS**				**Social satisfaction**				**Political satisfaction**			
			**Pre**	**Post**	***T*-test**		**Pre**	**Post**	***T*-test**		**Pre**	**Post**	***T*-test**
High	Income	Pers'l	0.003 (0.003)^NS^	0.012 (0.002)^***^	2.496	Com.	0.002 (0.002)^*^	0.004 (0.002)^*^	0.707	Nat'l	0.213 (0.023)^NS^	1.287 (0.293)^***^	3.654
	Trust	Pers'l	0.526 (0.038)^***^	0.506 (0.039)^***^	−0.367	Com.	0.054 (0.005)^***^	0.052 (0.005)^***^	−0.283	Nat'l	0.065 (0.034)^NS^	0.152 (0.032)^***^	1.863
	Interaction Trust*Income		0.0001 (0.001)^NS^	−0.002 (0.001)^**^	−1.485		0.0001 (0.000)^NS^	0.0000 (0.000)^NS^	0		0.011 (0.009)^NS^	0.013 (0.008)^NS^	0.166
	Variance—within		2.492/2.095	2.124/1.722			0.685/0.608	0.636/0.557			1.759/1.044	1.574/0.940	
	Variance—between		0.089/0.043	0.065/0.049			0.057/0.029	0.045/0.020			0.179/0.072	0.267/0.096	
	R^2^ within		0.159	0.189	18.8		0.112	0.124	10.5		0.406	0.544	33.8%
	R^2^ between		0.517	0.246	−52.4		0.491	0.556	13.1		0.598	0.640	7.1%
Medium	Income	Pers'l	0.016 (0.003)^***^	0.035 (0.005)^***^	3.258	Com.	−0.002 (0.002)^NS^	0.005 (0.002)^NS^	2.475	Nat'l	0.107 (0.539)^NS^	2.572 (0.349)^***^	3.839
	Trust	Pers'l	0.572 (0.039)^***^	0.709 (0.043)^***^	2.360	Com.	0.055 (0.004)^***^	0.058 (0.004)^***^	0.530	Nat'l	0.210 (0.050)^***^	0.190 (0.044)^***^	−0.300
	Interaction Trust*Income		−0.002 (0.001)^**^	−0.005 (0.001)^***^	−2.121		0.0000 (0.0001)^*^	0.0000 (0.0001)^NS^	0		−0.029 (0.014)^*^	−0.022 (0.012)^NS^	0.380
	Variance—within		4.036/3.449	4.101/3.367			0.938/0.810	0.798/0.689			2.370/1.477	2.214/1.306	
	Variance—between		0.479/0.294	0.285/0.201			0.083/0.070	0.056/0.010			0.383/0.231	0.763/0.259	
	R^2^ within		0.145	0.179	23.1		0.136	0.137	0.1		0.377	0.367	−2.6%
	R^2^ between		0.386	0.295	−23.7		0.157	0.821	424.5		0.397	0.661	66.4%
Low	Income	Pers'l	0.008 (0.012)^NS^	0.045 (0.016)^**^	1.850	Com.	−0.005 (0.003)^NS^	0.008 (0.004)^NS^	2.600	Nat'l	1.225 (0.138)^***^	0.742 (0.169)^***^	−2.214
	Trust	Pers'l	0.478 (0.050)^***^	0.599 (0.052)^***^	1.677	Com.	0.038 (0.003)^***^	0.036 (0.003)^***^	−0.471	Nat'l	0.079 (0.008)^***^	0.030 (0.027)^NS^	−1.740
	Interaction Trust*Income		0.002 (0.003)^NS^	−0.001 (0.004)^NS^	−0.600		0.0000 (0.0002)^NS^	0.0000 (0.0002)^NS^	0		0.011 (0.005)^*^	0.021 (0.008)^*^	1.060
	Variance—within		4.678/4.172	4.902/4.235			0.711/0.649	0.832/0.765			2.548/1.689	2.646/1.792	
	Variance—between		1.463/0.924	1.682/0.964			0.101/0.077	0.096/0.088			1.263/0.210	0.443/0.223	
	R^2^ within		0.108	0.136	25.8		0.351	0.081	−77.1		0.337	0.323	4.1%
	R^2^ between		0.368	0.427	15.9		0.238	0.083	−64.9		0.834	0.497	40.4%

*The trust–income interaction is also included as a test for the moderation of trust.*

*High: before N = 8,350; after N = 8,431. Medium: before N = 8,453; after N = 9,129. High: before N = 6,256; after N = 7,388. Analyses of satisfaction parameters were, in addition, controlled for gender, age, age^2^, marital status, number of people in a household, mental health, being sick or disabled, unemployed, educational level, and occupational level. Personal income is measured as yearly household income; community income per thousand is measured aggregated mean of household income for country, region, and social class; national income is measured as Ln GDP (PPP) per capita per thousand. Ranges of income: Personal income, −4.45–200; community income, −3–174; National income, 2.4–4.2; Ranges of trust: personal, 1–9; social, 0–30; political, 0–50. Ranges of satisfaction: social satisfaction, 0.3–173; political satisfaction, 0–10; life satisfaction (LS), 0–10. Levels in multilevel: country, region, and social class. Random intercept. Restricted maximum likelihood. Residuals weighted for post-stratification weight. Significance:*

* *p < 0.05;*

** *p < 0.01;*

*** *p < 0.001. NS = Not significant*.

In the micro layer, the relationship between personal LS and personal income from before to after the crisis was substantially and significantly strengthened in all three groups. In the high and low trust groups, the relationship went from not significant to clearly significant. In the medium trust group, the association was doubled. Even though the direct association of trust with personal LS was clearly significant both before and after the crisis in all three groups, and of the same general strength in the high and medium, but greater strength in the low trust groups; the difference in the relationship between personal LS and personal trust was strengthened only in the medium trust group. As indicated by the interaction (interaction Trust ^*^ Income), the modifying function of trust was significant before the crisis only in the medium group, whereas it was a significant modifier in the high and medium groups after the crisis. The negative, modifying function of trust was significantly strengthened, that is it became more negative, but only in the medium group.

In the mezzo layer, the association between social satisfaction and community income was only weakly significant in the high trust group but did not substantially change from before to after the crisis. The relationship between social satisfaction and social trust was strongly significant both before and after the crisis and remarkably stable from before to after the crisis in all groups. There was no modifying function of trust with community income, either before or after the crisis (interaction SocTrust ^*^ ComIncome) except in the medium group before the crisis.

In the macro layer, the association between political satisfaction and national income was significantly strengthened from before to after the crisis in the high and medium trust groups but was significantly weakened in the low trust group. In the medium trust group, it was highly significant after the crisis, but not before. The association between political satisfaction and political trust was only significant after the crisis in the high trust group, significant both before and after in the medium trust group, but the difference between was not significant, and only significant before the crisis in the low trust group. The modifying effect of trust was not or only weakly significant in all three groups, and the difference from before to after the crisis was not significant in any group (interaction trust ^*^ income).

The pseudo R^2^ provides information concerning the percent of variation explained by the model, both within and between countries. R^2^ values within countries indicate how much of the variance within each country is explained by the model being tested. Similarly, the R^2^ values between countries indicate how much the current model explains the variance between countries. Interesting changes in the pseudo R^2^ occurred.

In the micro layer, there was a rather substantial increase in the explained variance especially within countries in all three groups. However, between countries in the high and medium trust groups, there was a substantial drop in R^2^ from before to after the crisis, while in the low trust group R^2^ increased. This indicates that after the financial crisis, personal income and personal trust explained more of the variation in the personal LS values between countries in the low trust groups, but they explained less of the variation in the medium and high trust groups. Within countries, these variables explained more of the variance after the crisis for all three groups.

In the mezzo layer, there was an increase in pseudo R^2^ within countries in the high trust group, no change in the medium, and a substantial fall in the low trust group. Similarly, the R^2^ between countries showed an increase in the high and especially medium trust groups and a substantial fall in the low trust group. This indicates that after the financial crisis, community income, and social trust explained more of the variation in the social satisfaction values between countries in the low trust groups, but they explained less of the variation in the medium and high trust groups. Within countries, these variables explained more of the variance after the crisis for the high trust groups and explained less for the low trust countries.

In the macro layer, there was a substantial increase in pseudo R^2^ within countries in the high trust group, with little change in the medium and low trust countries, whereas there was a substantial increase in R^2^ between countries in the medium and low trust countries. This indicates that after the financial crisis, national income, and political trust explained more of the variation in political satisfaction values between countries in the medium and low trust groups. Within countries, these variables explained more of the variance after the crisis in the high trust countries.

### Results of Layers and Themes on LS

To examine the relationship between the community layer and the country layer in the individual layer, a full model including all the parameters in all layers was used. The results are shown separately for before and after the crisis, within each trust group of countries, in Table 4 and Figures 1, 2, together with significant testing of the changes from before to after the crisis.

**Table 4:** Unstandardized beta in a multilevel linear relationship of personal LS, before and after the financial crisis of 2008/2009, as a function of demographic, income, and trust parameters in each of the three groups of countries stratified by an overall trust.

**Country group**		**Before FC**			**After FC**				
		**Beta**	**SE**	**Sig**.	**Beta**	**SE**	**Sig**.	***T*-test**	**% change**
High	Personal trust	**0.488**	**0.021**	**0.000**	**0.382**	**0.020**	**0.000**	**−3.599**	**−21.7**
	Social trust	0.056	0.004	0.000	0.056	0.004	0.000	−0.132	−1.2
	Political trust	0.020	0.002	0.000	0.015	0.002	0.000	−1.893	−26.7
	Personal income	0.013	0.013	0.321	−0.006	0.015	0.705	−0.930	−145.0
	Community income	−0.010	0.028	0.720	0.019	0.025	0.436	0.782	289.1
	National income	−0.340	0.399	0.394	0.092	0.358	0.797	0.807	127.1
	ComInc*PersInc	0.000	0.000	0.420	0.000	0.000	0.000	−1.713	−282.4
	NatInc*PersInc	−0.002	0.004	0.529	0.004	0.004	0.319	1.163	278.0
	NatInc*ComInc	0.003	0.008	0.730	−0.005	0.006	0.452	−0.746	−280.8
Medium	Personal trust	**0.450**	**0.025**	**0.000**	**0.518**	**0.025**	**0.000**	**1.965**	**15.3**
	Social trust	0.060	0.004	0.000	0.065	0.004	0.000	0.875	8.6
	Political trust	0.026	0.002	0.000	0.030	0.002	0.000	1.127	13.9
	Personal income	0.045	0.022	0.038	0.105	0.048	0.028	1.147	133.9
	Community income	0.015	0.062	0.809	−0.059	0.079	0.456	−0.736	−492.7
	National income	−0.765	0.800	0.339	−0.490	0.528	0.354	0.287	35.9
	ComInc*PersInc	0.000	0.000	0.000	0.000	0.000	0.364	0.650	43.7
	NatInc*PersInc	−0.009	0.006	0.127	−0.024	0.013	0.064	−1.020	−156.5
	NatInc * ComInc	−0.003	0.018	0.880	0.015	0.021	0.481	0.641	669.2
Low	Personal trust	**0.465**	**0.033**	**0.000**	**0.567**	**0.030**	**0.000**	**2.276**	**21.9**
	Social trust	0.052	0.004	0.000	0.041	0.004	0.000	−1.725	−20.6
	Political trust	0.037	0.003	0.000	0.037	0.002	0.000	−0.157	−1.5
	Personal income	**0.099**	**0.030**	**0.001**	**0.688**	**0.121**	**0.000**	**4.711**	**593.9**
	Community income	**0.087**	**0.054**	**0.112**	**0.772**	**0.175**	**0.000**	**3.736**	**791.0**
	National income	**1.194**	**0.220**	**0.000**	**4.012**	**0.351**	**0.000**	**6.795**	**235.9**
	ComInc*PersInc	0.000	0.000	0.006	−0.001	0.000	0.061	−1.190	−189.0
	NatInc*PersInc	**−0.026**	**0.010**	**0.006**	**−0.193**	**0.037**	**0.000**	**−4.328**	**−633.1**
	NatInc*ComInc	**−0.020**	**0.017**	**0.242**	**−0.221**	**0.052**	**0.000**	**−3.656**	**−1008.8**

*Weighted N Before = 23,642; After = 25,060. Analyses controlled for gender, age, age^2^, marital status and number of people in a household, mental health, being sick or disabled, unemployed, educational level, and occupational level. Personal income is measured as yearly household income; community income per thousand is measured aggregated mean of household income for the country, region, and social class; national income is measured as Ln GDP (PPP) per capita per thousand. Ranges of income: personal income, −4.45–200; community income, −3–174; national income, 2.4–4.2; Ranges of trust: personal, 1–9; social, 0–30; political, 0–50. Ranges of satisfaction: social satisfaction, 0.3–173; political satisfaction, 0–10; LS, 0–10. Levels in multilevel: country, region, and social class. Random intercept. Restricted maximum likelihood. Residuals weighted for post-stratification weight. Significance p ≤ 0.05 is in bold*.

Within each group of countries from before to after the crisis the three trust variables were highly significant and independent parameters. However, in the high trust group, none of the three income variables were significant. In the medium trust group, only personal income was significant. In the low trust group, personal income and national income were significant before and all the three income variables were significant after the crisis. This indicates that in high trust countries, all forms of trust are important contributors to LS, but that income is not. In low trust countries, not only is trust very important to LS, but personal income and national income are quite important as well.

In the high and medium trust groups, only the relationship with personal trust changed significantly from before to after the crisis. In the high trust group, the relationship was weakened, while in the medium trust group, the relationship was strengthened. In the low trust group, there were significant increases in the associations for both personal trust and all the three forms of income, indicating that the importance of income for personal LS increased in the low trust countries after the crisis, a feature that did not happen with the high trust countries.

Figure 1 shows the relative effect of each variable on personal LS from before to after the crisis, calculated based on the weighted means for the population of each trust group and for before and after the crisis and the coefficients described in Table 4. As shown in Figure 1, the overall trend from before to after the crisis is a remarkably substantial increase in the relative importance of the income variables to the detriment of the trust variables.

A similar trend, although not so dramatic, was found between the groups, with an increase in the importance of the income variables from the high to the low trust group. Especially, social trust decreased in relative importance from high to low from before to after the crisis.

In the high trust group, the relative effect of personal trust more or less disappeared, as did that of social and political trust. These findings are partially confirmed in Table 4. National income went from a negative association before the crisis to a strong positive association after the crisis.

In the medium trust group too, the relative effect of the three trust variables vanished from before to after the crisis. The negative relative effect with national income increased substantially, while the effect estimate with community income went from weakly positive to strongly negative. The relative importance of personal income was almost halved after the crisis as compared to before the crisis.

In the low trust group too, the relative effect of the three trust variables was decreased, but not as substantially as seen in the high and medium trust groups. The relative importance of national income was positive and similar in both situations, whereas in the relative importance of community income, we found a sharp increase. Most of these findings are confirmed in Table 4.

Figure 2 shows the relative effect of the layers in each group of countries from before to after the crisis. Before the crisis, the micro layer decreased in importance from the high trust to the low trust group. The mezzo layer was the most important in the medium trust group, and the macro layer increased in importance from the high to the low trust group.

After the crisis, this situation was substantially changed. The macro layer became the most important in all the three trust groups. The most striking result, however, was the dramatic increase in the importance of the macro layer for all the three trust groups to the detriment of the micro layer.

Table 4 also shows the significance of the layer effect in each group of countries before and after the crisis. The interactions between national income and community or personal income and the association between community and personal income are indicative of the significance of the association of the layers on each other (Schyns, 2002).

In the high trust group, there were no significant layer effects of macro on mezzo and micro or mezzo on micro. After the crisis, the mezzo-micro relationship was found significant.

In the medium trust group, there was a significant macro-micro relationship before the crisis that was no longer there after the crisis. In the low trust group, there was a strong and significant macro-micro relationship that was six times stronger after the crisis. Similarly, the macro-mezzo relationship was almost 10 times stronger after the crisis.

### Moderation Analyses

Moderation was tested in two fashions, by adding the interaction term in the multilevel regression analysis (Table 3) and by using PROCESS (Table 5; X = personal income, Y = LS) (Table 6; X = national income and Y = political satisfaction).

**Table 5:** Results of the analysis for the moderator role of personal, social, and political trust for X = personal income and Y = LS, separated by before and after the financial crisis of 2008/2009 and country group as defined by overall trust.

**Country group**	**Moderator**		**Before**			**After**		
	**Trust**	**Levels of moderator**	***N***	**Effect on X(SE)Sig**	**R^**2**^/Interaction-sign**	***N***	**Effect on X(SE)Sig**	**R^**2**^/Interaction-sign**
High	Personal	Low	9,457	0.037(0.005)^***^	0.174/+0.002^NS^	9,089	0.070(0.005)^***^	0.193/−0.013^***^
		Medium		0.040(0.004)^***^			0.055(0.004)^***^	
		High		0.042(0.005)^***^			0.040(0.006)^***^	
	Social	Low	9,423	0.044(0.005)^***^	0.166/−0.010^***^	9,062	0.061(0.005)^***^	0.196/−0.012^***^
		Medium		0.032(0.004)^***^			0.046(0.004)^***^	
		High		0.021(0.005)^***^			0.033(0.004)^***^	
	Political	Low	9,147	0.043(0.005)^***^	0.152/−0.011^***^	8,845	0.058(0.005)^***^	0.180/−0.013^***^
		Medium		0.030(0.004)^***^			0.044(0.004)^***^	
		High		0.017(0.005)^**^			0.030(0.006)^***^	
Medium	Personal	Low	9,450	0.085(0.006)^***^	0.174/−0.013^***^	10,029	0.077(0.006)^***^	0.185/−0.020^***^
		Medium		0.069(0.005)^***^			0.053(0.004)^***^	
		High		0.053(0.006)^***^			0.029(0.006)^***^	
	Social	Low	9,388	0.086(0.006)^***^	0.184/−0.022^***^	9,994	0.069(0.006)^***^	0.201/−0.015^**^
		Medium		0.058(0.005)^***^			0.049(0.004)^***^	
		High		0.029(0.006)^***^			0.029(0.005)^***^	
	Political	Low	9,137	0.080(0.006)^***^	0.176/−0.014^***^	9,706	0.060(0.006)^***^	0.193/−0.013^***^
		Medium		0.062(0.005)^***^			0.043(0.004)^***^	
		High		0.045(0.006)^***^			0.027(0.006)^***^	
Low	Personal	Low	8,272	0.153(0.009)^***^	0.207/0.000^NS^	9,141	0.237(0.011)^***^	0.233/−0.008^NS^
		Medium		0.154(0.006)^***^			0.227(0.008)^***^	
		High		0.154(0.008)^***^			0.216(0.010)^***^	
	Social	Low	8,031	0.176(0.009)^***^	0.212/−0.018^***^	8,993	0.259(0.011)^***^	0.228/−0.024^***^
		Medium		0.152(0.006)^***^			0.226(0.008)^***^	
		High		0.128(0.008)^***^			0.194(0.010)^***^	
	Political	Low	7,531	0.171(0.009)^***^	0.227/−0.028^***^	8,543	0.266(0.010)^***^	0.243/−0.047^***^
		Medium		0.135(0.007)^***^			0.216(0.008)^***^	
		High		0.099(0.008)^***^			0.161(0.010)^***^	

*The analyses were performed using Hayes PROCESS, which does not allow for multilevel.*

*Analyses of satisfaction parameters were, in addition, controlled for gender, age, age^2^, marital status, number of people in a household, mental health, being sick or disabled, and unemployed. Personal income is the yearly household income measured as percentiles in 20 categories; community income per thousand is measured aggregated mean of household income for the country, region, and social class measured as percentiles in 20 categories; national income is measured as Ln GDP (PPP) per capita per thousand, measured as percentiles in 20 categories. Ranges of trust: personal, 1–5; social, 1–5; political, 1–5. Ranges of satisfaction: political satisfaction, 0–10; LS, 0–10. Residuals weighted for post-stratification weight. Significance:*

* *p < 0.05;*

** *p < 0.01;*

*** *p < 0.001. NS = Not significant*.

**Table 6:** Results of analysis for the moderator role of personal, social, and political trust for X = national income and Y = political satisfaction, separated by before and after the financial crisis of 2008/2009 and country group as defined by overall trust.

**Country group**	**Moderator**		**Before**			**After**		
	**Trust**	**Moder-ator levels**	***N***	**Effect on X(SE)Sig**	**R^**2**^/Interaction-sign**	***N***	**Effect on X(SE)Sig**	**R^**2**^/Moderator sign**
High	Personal	Low	9,574	−0.096(0.014)^***^	0.065/−0.025^**^	9,598	0.142(0.020)^***^	0.054/−0.057^***^
		Medium		−0.126(0.011)^***^			0.074(0.014)^***^	
		High		−0.156(0.015)^***^			0.006(0.020)^NS^	
	Social	Low	9,553	−0.081(0.015)^***^	0.144/−0.033^***^	9,579	0.141(0.019)^***^	0.132/−0.048^***^
		Medium		−0.121(0.010)^***^			0.084(0.013)^***^	
		High		−0.158(0.013)^***^			0.031(0.018)^NS^	
	Political	Low	9,377	−0.031(0.012)^*^	0.131/−0.022^**^	9,414	0.086(0.017)^***^	0.329/−0.013^NS^
		Medium		−0.069(0.009)^***^			0.072(0.012)^***^	
		High		−0.105(0.012)^***^			0.059(0.016)^***^	
Medium	Personal	Low	11,193	0.158(0.015)^***^	0.062/+0.002^NS^	12,331	0.111(0.014)^***^	0.061/+0.022^**^
		Medium		0.160(0.010)^***^			0.138(0.009)^***^	
		High		0.163(0.013)^***^			0.165(0.012)^***^	
	Social	Low	11,130	0.162(0.014)^***^	0.184/−0.022^***^	12,301	0.161(0.013)^***^	0.131/−0.026^***^
		Medium		0.133(0.009)^***^			0.127(0.009)^***^	
		High		0.104(0.014)^***^			0.092(0.012)^***^	
	Political	Low	10,905	0.132(0.012)^***^	0.371/−0.005^NS^	12,071	0.062(0.009)^***^	0.433/−0.008^NS^
		Medium		0.126(0.008)^***^			0.052(0.008)^***^	
		High		0.120(0.011)^***^			0.042(0.012)^***^	
Low	Personal	Low	9,448	0.517(0.024)^***^	0.124/−0.007^NS^	11,017	0.217(0.022)^***^	0.043/−0.033^**^
		Medium		0.508(0.016)^***^			0.174(0.016)^***^	
		High		0.499(0.022)^***^			0.131(0.022)^***^	
	Social	Low	9,263	0.536(0.022)^***^	0.196/−0.035^**^	10,873	0.209(0.021)^***^	0.105/−0.048^***^
		Medium		0.489(0.016)^***^			0.146(0.015)^***^	
		High		0.442(0.022)^***^			0.083(0.023)^***^	
	Political	Low	8,922	0.274(0.021)^***^	0.385/−0.002^NS^	10,525	0.117(0.019)^***^	0.292/−0.039^**^
		Medium		0.271(0.015)^***^			0.075(0.014)^***^	
		High		0.268(0.018)^***^			0.030(0.020)^NS^	

*The analyses were performed using Hayes PROCESS, which does not allow for multilevel.*

*Analyses of satisfaction parameters were, in addition, controlled for gender, age, age^2^, marital status, number of people in a household, mental health, being sick or disabled, and unemployed. Personal income is the yearly household income measured as percentiles in 20 categories; community income per thousand is measured aggregated mean of household income for country, region, and social class measured as percentiles in 20 categories; national income is measured as Ln GDP (PPP) per capita per thousand, measured as percentiles in 20 categories. Ranges of trust: personal, 1–5; social, 1–5; political, 1–5. Ranges of satisfaction: political satisfaction, 0–10; LS, 0–10. Residuals weighted for post-stratification weight. Significance:*

* *p < 0.05;*

** *p < 0.01;*

*** *p < 0.001. NS = Not significant*.

Table 5 presents the results of moderation analyses for X–Y relationship personal income, LS. First, the significance of the moderation is tested by confirming the significance of the interaction term. If the significance is verified, the significance of the effect at different levels (low, medium, and high) of the X parameter is then tested. These results are summarized in Tables 5, 6.

Personal trust acted as a moderator in the high trust group, but only after the crisis. In the medium trust group, the personal trust acted as a moderator both before and after the crisis. In the low trust group, it did not act significantly as a moderator either before or after the crisis. The interaction effects, when significant, were always negative, indicating that personal trust moderated by decreasing the slope of the relationship, or as a buffer (c.f. “the buffer hypothesis”). When significant, the effect was also significant at all three levels of X.

Social trust was a negative moderator in all three country groups both before and after the crisis. The general trend was that the size of the interaction effect with social trust increased from the high to the low trust group. In the high and low trust countries, the size of the interaction effect also increased from before to after the crisis. In the medium trust countries, however, it decreased. Again, the interaction effects were negative. This indicates that the level of the interaction effects decreased as the level of trust increased. Again, the effect size was significant for all three levels of X.

Political trust was a negative moderator in all three country groups both before and after the crisis. As was the case with social trust, the size of the interaction effect of political trust increased from the high to the low trust group. Additionally, the size of the effect increased from before to after the crisis, but only in the low trust group. In the high and medium trust groups, it remained essentially the same from before to after the crisis. As previously described for social trust, the effect estimates were significant for all three levels of X.

Table 6 presents the results of the moderation analyses for X–Y relationship national income-political satisfaction.

In the high trust group, all three forms of trust were significantly negative moderators before the crisis. After the crisis, however, the only personal and social trust acted as moderators. Since these represent values for centered data, they can be compared. Before the crisis, the strongest moderator was social trust. After the crisis, however, personal trust was the strongest moderator. For all significant interactions, the effect estimates were significant at all three levels of X.

In the medium trust group, personal trust was only a significant moderator after the crisis. Social trust was negative and of the same relative magnitude both before and after the crisis. Political trust was not significant in either situation. For all significant interactions, the effect estimates were significant at all three levels of X.

In the low trust group, only social trust was significant before the crisis. After the crisis, all three forms of trust were significant negative moderators. For all significant interactions, the effect estimates were significant at all three levels of X.

## Discussion and Conclusion

The financial crisis of 2008/2009 not only impacted the European countries very differently, but the governments also handled the crisis in very different ways (Davies et al., 2010; Karanikolos et al., 2013; OECD, 2014). Therefore, it is not surprising that countries differed also in the impact the crisis had on both the personal well-being of their population and satisfaction with their national government.

In a previous article, we have shown that personal trust moderates the relationship between personal income and LS. We have also shown that social trust moderates the relationship between community income and social satisfaction and that political trust moderates the relationship between national income and political satisfaction. However, when European countries were grouped according to their level of social and political trust, the close associations between LS and personal, community and national income were only evident in low-trust countries. Trust, thus, seemed to buffer the effect of income on personal LS in medium and high-trust countries (Clench-Aas and Holte, 2021).

This article takes the “the buffer hypothesis” one step further by asking: might the buffering capacity of trust protect against decreased well-being in a population when faced with a national or global crisis? To address this important question, we used the theoretical model of Dahlgren and Whitehead as the point of departure. This model places the individual at the center of several nested, hierarchically organized socio-structural layers, namely, the individual, community, and country (Clench-Aas and Holte, 2021). The unique aspect of this model is that each socio-structural layer has its layer-specific income, trust, and satisfaction parameters. We additionally divided the European countries into three groups according to their levels of social and political trust.

The results are strongly consistent with the “the buffer hypothesis.” Overall, in countries with a high level of social and political trust before the financial crisis, the crisis did not influence trust or satisfaction levels severely. In countries with low levels of trust, however, the crisis was followed by a severe decline in both trust and satisfaction levels. We also found that national factors, such as political trust and national income, were more important for personal LS after the crisis. This suggests that strong national measures may be effective mechanisms to strengthen well-being within the country.

### Aim 1: To Assess How the Relationship Between Income and Satisfaction Within Different Layers of the Society When Accounting for Personal, Social, and Political Trust Changed From Before to After the Financial Crisis of 2008/2009

We examined the effect of the crisis on the layers in two ways: (1) changes in absolute level and (2) changes in the strength of the associations between layer-specific satisfaction, income, and trust.

The micro layer was characterized by lower absolute levels of personal income in the low and medium trust countries, slightly increased levels of personal trust, and increased personal LS in all three groups. The association between LS and personal income was greater after the crisis, especially in the low and medium trust groups. This suggests that improvements in personal income led to improvements in personal LS after the crisis. The decline in personal LS following the financial crisis, observed in both Europe and America, did not last long and was followed by increased personal LS (Deaton, 2012; Clench-Aas and Holte, 2017).

The mezzo layer was characterized by increased levels of social satisfaction despite declines in community income in all the three trust groups. The levels of social trust were remarkably similar before and after the crisis. Despite the large declines in community income in the medium and low trust groups, the relationship between community income and social satisfaction was substantially strengthened in these two groups. A greater positive association indicates that not only does social satisfaction increase with higher community income but that it may also indicate that in the poorest communities, social satisfaction was reduced. The association of social trust was again remarkably stable. Overall, the results from the mezzo layer underscore the importance of the local economy for social satisfaction. This may possibly serve as an explanation for the restoration of higher levels of social satisfaction after the crisis.

In the macro layer, even though national income increased from 2006 to 2012, there were clear drops in GDP during the crisis, yet some transition countries were less influenced and retained an increasing trend (Clench-Aas and Holte, 2017). Overall, the differences between the high and low trust countries were most evident at the macro level, with clear increases in political trust and satisfaction in the high trust countries and substantial declines in the low trust countries. The relationship between national income and political satisfaction was substantially strengthened in the high and medium trust nations. In the low trust countries, however, it was substantially weakened. The trend was similar but not significant for political trust and political satisfaction. This is an important finding since some of the transition countries were in a period of economic growth during this period, yet the trust in and the satisfaction with authorities were weakened. This layer was the least robust and exhibited clear challenges from the financial crisis.

#### Does the Literature Support These Findings?

The literature that discusses the overall layer effects is limited! However, some studies have investigated individual links.

Pooled data of 27 EU member states from the Eurobarometer survey of 2011 indicate a slight decrease in trust in the European Union, the national parliament, and the national government during the financial crisis with a sharp decline over 6 months from spring 2011 (European Commision, 2011). There are also other studies pointing to reduced levels of both social and political trust after the financial crisis (Stevenson and Wolfers, 2011; Papaioannou, 2013; Habibov and Afandi, 2015; Darvas and Wolff, 2016; Bartolini et al., 2017; Navarro-Carrillo et al., 2018; Ananyev and Guriev, 2019).

However, the results indicate that there is a strong need to differentiate such results according to the level of trust before the crisis. For instance, in Finland, a high trust country, high social trust was observed during their 1990 economic crisis despite decreasing levels of trust in the political and public institutions (Newton, 2001). Newton (2001) argues that this was due to the political origin of the problems, which were unrelated to other factors that could contribute to decreasing social trust. Although in this study we observed a slight fall in social trust from before to after the crisis, we do confirm high levels of social trust in Finland.

A significant increase in social trust between 1999 and 2010 was also reported in another high trust country, Iceland, during their financial crisis (Growiec et al., 2012). The authors suggest that this increase, as well as a corresponding increase in sociability, reflects a coping response to the stress caused by the crisis.

### Aim 2: To Assess if Countries, Grouped According to Their Levels of Trust, Differ in the Importance of the Financial Crisis on LS

The total effect of the financial crisis on personal LS was quite different in the three trust groups.

In the high trust group, there were minor changes in LS. This may indicate that the high trust levels served as a buffer against the crisis (even though the moderating role of the three trust forms was lower than in the other groups). After the crisis, however, there were indications that the protective effect of trust may have diminished since the relative importance of all the three forms for income increased, while the protective role of personal trust increased.

In the medium trust group, there were similar trends, although both the association between trust and personal LS and the moderation was stronger than in the high trust groups, similarly, the importance of the income variables rose. Again, the dominant change after the crisis was toward greater importance of personal trust (i.e., “self-efficacy”).

The most dramatic changes occurred in the low trust group. Here, there was little to indicate that trust acted as a buffer despite an increase in the moderator role of social and political trust. Quite the contrary, the importance of the income variables increased dramatically after the crisis, as did the association with personal trust.

### Aim 3: Holistically, to Determine the Relative Importance of the Financial Crisis to Individual LS, After Accounting for All Variables of Income and Trust at Each Layer, i.e., Individual, Community, and Country

#### The Relative Importance of Trust and Income on LS

The results differed substantially among the three trust groups. In the high trust group, which is dominated by the Nordic countries, the relationship between LS and all the trust variables either decreased or remained the same from before to after the crisis. The only significant decline was indicated for personal trust. None of the income variables were significantly associated with personal LS either before or after the crisis.

Using these associations to determine relative importance reveals that before the crisis, the relative importance of trust was very large in the high trust group. Altogether, 60% of the relative effect of the three income and three trust variables on personal LS could be attributed to one form or another of trust. After the crisis, the relative importance of trust fell to 5.5%. The relative importance of national income changed from −21.6 to +39.1% (Figure 1).

In the medium trust group, of all the trust variables, only personal trust changed its association with personal LS from before to after the crisis, thereby increasing the strength of the association. The association between personal income and personal LS increased, but the change was not significant. Combining the associations with the measured levels of the parameters led to an increase in the relative importance of the income variables, although less dramatic than in the high trust group. The increased relative importance of national income, however, was high, from −33.8 to +54.5 (Figure 1).

In the low trust group, we observed a significant increase in the association between personal trust and personal LS after the crisis. However, the low trust group distinguished itself from the other two groups in terms of the importance of the income variables, with the associations showing dramatic increases from before to after the crisis. The resulting relative effect was less dramatic although in the same direction (i.e., the increased effect of the income variables and reduced effect of the trust variables).

These findings suggest that as national income increases, the ability to use public funding to aid the population, for example, in terms of improved unemployment measures and aid to families, etc., may have a greater impact on the general well-being.

#### The Importance of the Outer Layers on the Individual

All three trust groups exhibited a clear increase in the importance of the outer or macro layer at the expense of the micro layer. This was, however, most prominent in the high and medium trust groups.

These findings were, however, not confirmed in the statistical test of the layer effect, using interactions. In the high and medium trust groups, from before to after the crisis, there were none or very modest significant layer effects on personal LS, and consequently no significant changes in these effects.

More surprising was the very sharp increase in the importance of the layer effect, as measured by the interaction term, between the national and community and personal layers in the low trust group. Therefore, even though what happens in the country (i.e., national) layer is far more important to personal LS than what happens in the community or individual layer, the relative importance of these latter two layers increased from before to after the crisis.

The dramatic increase in the relative importance of the macro or national layer to the detriment of the micro level is an important finding and provides the governments, especially in low trust countries, with more influence, increasing their ability to initiate measures that can lead to positive changes in well-being for the country as a whole.

#### Does the Literature Support These Findings?

There is less consensus on the association between national income and well-being, and as to which form of income is the most important (Biswas-Diener, 2008; Caporale et al., 2009; Diener et al., 2013, 2018). The role of community income is more complex and seems to be related to the size of the community, with larger communities showing negative associations between well-being and community income (Blanchflower and Oswald, 2004; Kingdon and Knight, 2007; Barrington-Leigh and Helliwell, 2008; Graf and Tillé, 2013; Brodeur and Flèche, 2018). At the neighborhood level, most studies have reported positive associations (Kingdon and Knight, 2007; Barrington-Leigh and Helliwell, 2008; Knies et al., 2008; Clark et al., 2009; Dittmann and Goebel, 2010; Brodeur and Flèche, 2018; Ma et al., 2018). However, the role of a global crisis on the association between the different income forms and well-being is less documented.

The positive association between trust and well-being has frequently been reported (Helliwell and Huang, 2008; Helliwell and Wang, 2010; Bartolini et al., 2013b; Helliwell et al., 2016). However, the relative importance of the different forms of trust is sparsely studied, and there is less agreement (Mota and Pereira, 2008; Bartolini et al., 2013a). Finally, little is known as to the effects of an economic crisis on these associations. The decreased association between political trust and personal LS after the crisis may also result from a general decrease in political trust over the years (Catterberg and Moreno, 2005; Blind, 2007).

Our results do not support previous findings indicating that social trust is more closely related to well-being than national income (Bartolini and Sarracino, 2014). In fact, we found the reverse. One explanation may be that this previous study examined long-term effects, whereas the current study could only estimate short-term effects. Finally, although a close relationship between high self-esteem and well-being has been reported (Baumeister et al., 2003; Lyubomirsky et al., 2006; Margolis and Lyubomirsky, 2018), the greater relative importance of personal trust, as opposed to the other forms of trust, has not been reported.

There is some support for the observed layer effect. Our findings are partially consistent with previous findings as to the importance of political satisfaction, political trust, and national income on personal LS had been well-documented across countries (Schyns, 2002; Morrison et al., 2011; Reeskens and Wright, 2011; Helliwell et al., 2014a, 2018). Additionally, it has been observed that political trust and political satisfaction are more important than social trust in predicting personal LS (Mota and Pereira, 2008).

The importance of the community layer on personal well-being has been described previously. The direction of the effect seems to be dependent on the size of the geographic area, for example, neighborhoods vs. larger geographical units (Kingdon and Knight, 2007; Barrington-Leigh and Helliwell, 2008; Brodeur and Flèche, 2018). There are indications that the financial crisis increased the reliance of individuals on themselves as the impact of events in a national layer increased as dominating forces to personal LS (Bjørnskov, 2003; Algan and Cahuc, 2014).

One of the few studies that examined this with respect to the financial crisis indicated that increased social support mitigated the negative consequences of the financial crisis in Portugal, a low trust country (Matavelli et al., 2020). Another study indicated that the financial crisis, by increasing financial worries, created difficulties in social identity and, hence, relationships with other individuals in their network (Heretick, 2013). However, this study is far more comprehensive than previous studies, as it includes all the three layers in the same model and the changes from before to after a global crisis.

### Aim 4: To Determine if the Eventual Buffering Role of Trust on the Relationship Between Income and Satisfaction Within Each Layer Holistically Changed After Exposure to the Financial Crisis, “the Buffer Hypothesis”

Moderation was examined in several different ways: (1) by calculating the interaction of the moderating variable and the explanatory variable, (2) by moderation testing using Hayes moderation and mediation program, and (3) by dividing the sample into levels of the proposed moderator and examining the relationships of the variables of interest within these layers. In addition, we examined the role of the three trust variables both within each layer and holistically upon personal LS and political satisfaction.

#### Moderating Effect of Trust Within Layers

There were important changes in the moderating capacity of trust in each layer. Personal trust acted as a buffer that became even stronger after the crisis within the micro layer in the high and medium trust groups but not in the low trust group. Likewise, social trust was not a significant modifier within the mezzo layer in any group. Political trust was only a modifier in the low trust countries, and its positive interaction did not change substantially after the crisis. Consequently, only personal trust showed a clear and measurable buffering effect within the three layers.

#### Moderating Effect of All Three Forms of Trust on LS

Using the Hayes model, all the three forms of trust in the high trust group showed small but, nevertheless, greater buffering effects from before to after the crisis. In the medium trust group, there was a substantial increase in the buffering role of personal trust, a reverse in social trust, while the buffering role of political trust remained unchanged. In the low trust group, all three forms of trust showed a substantially increased buffering capacity. The modifying role of all the three forms of trust was also much stronger than in the two other groups.

#### Moderating Effect of All Three Forms of Trust on Political Satisfaction

In view of the importance of political satisfaction for the satisfactory resolution or amelioration of difficulties caused by a major crisis, we chose to examine the modifying roles of trust on political satisfaction. Again, the moderating test within the Hayes model was used.

In the high trust group, only personal and social trust showed increased buffering effects from before to after the crisis. In the medium trust group, there was a substantial increase in the buffering role of personal trust, a minor increase in social trust, while the buffering role of political trust remained unchanged. In the low trust group, the results were in sharp contrast to those measured for LS. For political satisfaction, all the three forms of trust showed substantially decreased buffering capacity from before to after the financial crisis. However, the modifying role of all the three forms of trust was still much stronger than in the two other groups.

#### Moderating Effect of Trust on Personal LS by Examining Results by Groups Based on Levels of Trust

This has been discussed under Aim 2; but as a summary, in this study, we will mention that the potentially negative effects of the financial crisis, as measured through the association of the three forms of income on LS, were not observed in the high trust groups, had mixed results in the medium trust groups, and, clearly, evident in the low trust groups. This provides further confirmation of a moderator role for all the three forms of trust on LS, which increased in importance from before to after the crisis.

Self-esteem has an integral function in the social identity of the individual that helps place and strengthens the participation of individuals in a local or close network, be it family, friends, or community. It is closely related to the ability of people to communicate and be open with respect to others (Cast and Burke, 2002). Trust has previously been suggested as an important element for coping with crises, especially in transition countries (Helliwell et al., 2014a, 2016; Habibov and Afandi, 2015; Bartolini et al., 2017). Also, one study indicates that social support, which is linked to social trust, had a clear moderator function in Portugal, a low trust country, with respect to the negative effect of the financial crisis on personal LS (Matavelli et al., 2020). The results are consistent with such assumptions on a European basis, especially in the transition countries, which are the main constituent of the low trust group.

### Public Health Consequences and General Conclusions

This study enhances the current literature by investigating the holistic effects of global events on the individual. We did that by including three of the most important socio-structural layers we exist within, namely the individual, local community, and country. Global events do not similarly affect these layers. Consequently, it is not surprising that these events will impact individuals differently.

Trust has been called the Nordic gold (Andreasson, 2017). This claim is largely supported in this study. We found that trust is important in each of the three socio-structural layers; trust in oneself, trust in the local community of an individual, and trust in how the county is run. We have demonstrated the significance of trust in coping with crises, in this case, the financial crisis of 2008/2009. Importantly, we also showed trust to buffer the relationships between the different forms of income and satisfaction.

Being a repeated cross-sectional study, we can only speculate as to the causal mechanisms behind the significance of trust in times of crisis. We suggest, however, that the different forms of trust work as lubricants on the relationship between income and well-being for the individual, local community, and society at large (Clench-Aas and Holte, 2021), thus providing resilience when facing crises.

Trust simply makes life easier, simpler, more pleasant, and friendly. In public health, trust is strongly associated with individual happiness, altruistic attitudes, collaboration between people, a sense of control of the life of an individual, and better chances in life (Putnam et al., 1994; Cast and Burke, 2002; Rodríguez-Pose, 2012; Rothstein, 2013). Economically, trust is associated with fewer formalities, conflicts, legal processes, lower transaction costs in commerce, and favorable conditions for investment (Zak and Knack, 2001; De Groot et al., 2004; Tabellini, 2010). Politically, trust seems to promote political engagement and democratic development and reduce crime (Kennedy et al., 1998; Putnam, 2001).

In times of crisis, the trust may dissipate most of the negative consequences of the crisis, be they economic or otherwise. This buffering role of trust may possibly explain the relative stability of personal LS from before to after the crisis. This study underpins this view by showing how little the financial crisis of 2008/2009 was associated with changes in the high trust countries, yet how much it influenced the low trust countries in Europe.

We found that after the crisis, measures in the national layer increased in importance at the cost of measures in the community and individual layers for personal well-being. This may imply that following a global crisis, the actions taken by institutions at the country level become even more important.

Furthermore, we assume that societies with high levels of national or social trust may be able to more freely implement the necessary measures to tackle the event while maintaining the support of the population. When national policies are accepted, it is simpler for the community to also institute measures that will be accepted. The decrease in the negative reaction to necessary measures may create a positive spiral that allows continuing and furthering necessary measures.

This process is even more important when there are economic consequences of a global event. By allowing the collective well-being to be maintained, and even sometimes increased, the positive spiral can be further stimulated by increasing creativity and productivity, in that way aiding in the solution to the problems. Strengthened self-esteem and social identity lead to greater commitment and increased responsibility necessary to rebuild social structures that are often impaired during a global crisis (Cast and Burke, 2002).

Thus, this study lends support to the suggestion that politicians, professionals, and regulators have an important task in increasing their levels of population of all three forms of trust. This is especially important given the buffering role of trust on the impact of income on well-being in times of crisis. A challenge for future research, however, is to see whether the results from this study are also valid for other national or global crises, such as the COVID-19 pandemic.

## Strengths and Limitations

The major strength of this study is that we used a combination of multilevel analysis and a three-layer socio-structural model. This way, we could holistically assess how each layer-specific theme of income, trust, and satisfaction is independently associated with LS, controlling for all the others.

Another major strength is the large sample size obtained by ESS and their use of methodological standards in all stages of the process. This makes the data ideal for comparative and cross-national analyses. The ESS team is working continuously to ensure high validity and reliability of the questionnaire and data collected. The use of strict randomized probability sampling provides an assumingly representative sample of the population, and the questionnaire used is well-tested and translated according to ESS protocols.

The third strength is that we could utilize available and comparable EES data from both before (2006) and after (2012) the financial crisis of 2008/2009. Consequently, we were able to study longer-term relationships between income, trust, and satisfaction.

The fourth strength is the large number of relevant confounders included in the analyses, such as gender, age and age^2^, number of people in a household, marital status, education, occupation, unemployment, and mental health, which is the single most important contributor to variation in personal LS that can be explained by observables.

The fifth strength is that it includes comparable data across 19 European countries. This made it possible both to study the relative effect of the national layer as such and to come closer to theorizing about Europe as a whole. However, although more countries were included than in any previous similar studies, the after all limited number of countries limits generalization to all Europe. The inclusion of data from other countries was not available for the period examined in this study.

The sixth strength is that we were able to divide the 19 countries into three fairly equally sized, distinct groups according to their levels of overall social and political trust. This made it possible to discern the different relationships between income and personal LS in high trust countries vs. medium and low trust countries.

This study has several limitations, too. Although the surveys were conducted both before and after the financial crisis of 2008/2009 and with 6 years of interval (2006–2012), the cross-sectional nature of the data limits the possibility to draw causal conclusions from the findings. Even though these countries are grouped according to overall social and political trust levels, there is no doubt that these groups of countries also differ in other parameters.

Several of the measures were based on self-report, and thus response bias might be present. However, the main part of these measures, such as those on trust and satisfaction, is truly subjective measures and can hardly be measured validly by other methods than by asking people. Due to the novelty of the approach used in this study, there is a lack of literature concerning either the theory behind or the interpretation of the measures used for trust and well-being.

The items used to measure satisfaction and trust in all three layers could have been more consistent across layers. While satisfaction in the micro layer referred to personal LS as a whole, satisfaction in the mezzo layer was limited to areas of belonging, social support, respect, and safety; and satisfaction at the macro layer referred to satisfaction with how the country was run. Unfortunately, we did not have data on satisfaction with how the community was run, such as the local provision of public services. However, this may, to some extent, be compensated by the claim that the greatest effect on satisfaction with the community comes from how satisfied one is with social contacts of an individual.

Furthermore, we often used single-item questions to indicate the constructs examined. However, possible threats to the reliability of these measures are, to a large extent, compensated by the large sample size.

The ESS includes no standard measure of trust in oneself. However, although not 100% perfect, the concepts of self-confidence and self-esteem are logically very close. We, therefore, used self-esteem as a proxy for trust in oneself.

The data needed to determine measures on the community layer were only available for two rounds (3 and 6), resulting in a lower sample size. In the community layer, information about the neighborhood was not available. We, therefore, used regional level within countries to represent the local community. However, it has been shown that larger regional units represent more satisfactorily the community effect than estimates at the neighborhood level (Rickardsson and Mellander, 2017). To address this problem, we combined information on the regions within the country that the individuals lived in together with the social class. Although this was not ideal, it was considered an acceptable approximation. It cannot be denied that it would have been preferable with a more precise definition of neighborhood. Yet, the fact that we found a strong relationship within each layer, including the relationships between community income, respectively, and social satisfaction, may indicate that the way we defined local community worked out well.

Unfortunately, the income variable was changed in 2008 from 12 identical categories to 10, specific for each country. However, we controlled for this by imputing a personal income for each respondent using nation-specific information on the distribution by gender, age, and education. This allowed an acceptable form of harmonization between the two periods. It would have been preferable, however, that the true income had been provided for each of the years, but our approximation functioned well according to statistical properties. For further details, refer to Supplementary Material 2 - Table 3. It would have been preferable if we, in addition to income, representing money into a household, also had a measure of expenses, such as debt, household expenses, and so forth.

## Data Availability Statement

Publicly available datasets were analyzed in this study. This data can be found at: https://www.europeansocialsurvey.org/data/.

## Ethics Statement

The studies involving human participants were reviewed and approved by ESS ERIC. Written informed consent to participate in this study was provided by the participants' legal guardian/next of kin.

## Author Contributions

JC-A, IB, RN, and AH contributed to the conception and design of the work and contributed to the final approval of the version to be published. JC-A and IB were responsible for data analysis and interpretation as well as writing the first draft of the article. JC-A, IB, and AH contributed to further drafts and revisions of the article. All authors contributed to the article and approved the submitted version.

## Conflict of Interest

The authors declare that the research was conducted in the absence of any commercial or financial relationships that could be construed as a potential conflict of interest.
