# Supplementary material for: Trust Buffers Against Reduced Life Satisfaction When Faced With Financial Crisis
Source: Front Psychol. 2021 Jun 24;12:632585. doi: 10.3389/fpsyg.2021.632585 (PMC8264375; doi:10.3389/fpsyg.2021.632585)
Supplement: Supplementary file 1 [file Data_Sheet_1.docx]

**S1-Methods. Supplementary information on Materials and Methods**

**Level defining measure**

**Education**

Education was calculated based on the highest level of education attained and classified using the International Standard Classification of Education (ISCED) originally developed by UNESCO. The classification system resulted in the following categories: 1) Less than lower secondary education (ISCED 0-1); 2) Lower secondary education completed (ISCED 2); 3) Upper secondary education completed (ISCED 3); 4) Post-secondary non-tertiary education completed (ISCED 4); 5) Tertiary education completed (ISCED 5-6). This classification system was used to categorize the individual and the partner.

**Occupation**

Occupation was calculated using the script developed by Tafik and Oesch for ESS based on the concept by Oesch [[91](#_ENREF_91), [92](#_ENREF_92)]. The script constructs an indicator of occupation based on two dimensions. A first dimension is vertical and discriminates between more or less privileged employment relationships. The second dimension is horizontal and distinguishes between different work logics. The concept of work logic refers to differences between occupations in the potential for the division of labor, the type of skills required, or the nature of authority relations. The combination of the vertical and horizontal dimensions produces a 5-class schema based on an original 16-class. Occupation was defined by ISCO88 (2002 – 2010) or ISCO08 (2012 – 2014). This classification system was used to categorize the individual and the partner.

**Income**

*Personal income.* The respondent was given a show card that enabled them to choose between their weekly, monthly or annual income, whichever they found easiest, and there were 12 predetermined categories that were identical for all countries. As of 2008, a decile approach has been applied when measuring income in the ESS. The categories were national and based on deciles of the actual household income range in the given country. The deciles were documented in national currency. To harmonize these two different methods of defining income the following procedure was used:

*Personal income variable from 2006:* At first, the values for the 12 categories were replaced with the midpoint of the predetermined range for each of the 12 categories. The relationships between income and gender, age and education were calculated for each country. The resulting regression coefficients were used to adjust (impute) personal income either up or down from the midpoint, depending of the age, gender and education of each individual. More specifically, the relationship between personal income and age was not linear but an upside down U, while the relationships between personal income and gender, and income and education, both were linear.

*Personal income variable 2012*: The procedure was similar to that of 2006. However, each country now operated with deciles now appropriate for that country and that year. The midpoint and range of each of the 10 deciles, for each country in terms of yearly income in euros was calculated. Based on this midpoint, similar regression coefficients were calculated and the remaining procedures were identical to that of 2006.

*National income*. Data are in current international dollars (PPP) based on 2011 ICP round. An international dollar has the same purchasing power over GDP as the U.S. dollar has in the United States. GDP was downloaded from World Bank national accounts

For some analyses, the values were described as per 1000 PPS. PPS is the technical term used by Eurostat for the common currency in which national accounts aggregates are expressed when adjusted for price level differences using PPPs. Thus, PPPs can be interpreted as the exchange rate of the PPS against the [euro](http://ec.europa.eu/eurostat/statistics-explained/index.php/Glossary:Euro). For practical reasons of readability and interpretation of regression estimates, the values were divided by 1000.
