# Supplementary material for: Trust Buffers Against Reduced Life Satisfaction When Faced With Financial Crisis
Source: Front Psychol. 2021 Jun 24;12:632585. doi: 10.3389/fpsyg.2021.632585 (PMC8264375; doi:10.3389/fpsyg.2021.632585)
Supplement: Supplementary file 2 [file Data_Sheet_2.docx]

**SM2-Table1**: **Sample size and response rate for each country and for each study year.**

|  | 2006 |  | 2012 |  |
| --- | --- | --- | --- | --- |
|  | Sample size | % response rate | Sample size | % response rate |
| Belgium | 1798 | 61.0 | 1869 | 58.7 |
| Bulgaria | 1400 | 64.8 | 2260 | 74.7 |
| Cyprus | 995 | 67.3 | 1116 | 76.8 |
| Denmark | 1505 | 50.8 | 1650 | 49.1 |
| Finland | 1896 | 64.4 | 2197 | 67.3 |
| France | 1986 | 46.0 | 1968 | 52.1 |
| Germany | 2916 | 54.5 | 2958 | 33.8 |
| Ireland | 1800 | 56.8 | 2628 | 67.9 |
| Netherlands | 1889 | 59.8 | 1845 | 55.1 |
| Norway | 1750 | 65.5 | 1624 | 54.9 |
| Poland | 1721 | 70.2 | 1898 | 74.9 |
| Portugal | 2222 | 72.8 | 2151 | 77.1 |
| Russia | 2437 | 69.5 | 2484 | 67.0 |
| Slovakia | 1766 | 73.2 | 1847 | 74.1 |
| Slovenia | 1476 | 65.1 | 1257 | 57.7 |
| Spain | 1876 | 65.9 | 1889 | 70.3 |
| Sweden | 1927 | 65.9 | 1847 | 52.4 |
| Switzerland | 1804 | 51.5 | 1493 | 51.7 |
| United Kingdom | 2394 | 54.6 | 2286 | 53.1 |

**SM2-Table2. Number of missing data (M) together with total count (N) for variables of interest and for each country.**

|  | **LS** | | **Social Satisfaction** | | **Polt’l satisfaction** | | **Personal trust** | | **Social trust** | | **Polt’l trust** | | **Ln GDP** | | **Personal community income** | |
| --- | --- | --- | --- | --- | --- | --- | --- | --- | --- | --- | --- | --- | --- | --- | --- | --- |
|  | **N** | **M** | **N** | **M** | **N** | **M** | **N** | **M** | **N** | **M** | **N** | **M** | **N** | **M** | **N** | **M** |
| Belgium | 3667 | 4 | 3667 | 25 | 3667 | 131 | 3667 | 4 | 3667 | 6 | 3667 | 61 | 3667 | 0 | 3667 | 412 |
| Bulgaria | 3660 | 23 | 3660 | 177 | 3660 | 821 | 3660 | 88 | 3660 | 125 | 3660 | 366 | 3660 | 0 | 3660 | 583 |
| Switzerland | 3297 | 3 | 3297 | 78 | 3297 | 326 | 3297 | 6 | 3297 | 17 | 3297 | 277 | 3297 | 0 | 3297 | 622 |
| Cyprus | 2111 | 6 | 2111 | 98 | 2111 | 306 | 2111 | 11 | 2111 | 34 | 2111 | 160 | 2111 | 0 | 2111 | 415 |
| Germany | 5874 | 6 | 5874 | 100 | 5874 | 440 | 5874 | 34 | 5874 | 24 | 5874 | 215 | 5874 | 0 | 5874 | 1449 |
| Denmark | 3155 | 10 | 3155 | 141 | 3155 | 182 | 3155 | 34 | 3155 | 28 | 3155 | 123 | 3155 | 0 | 3155 | 423 |
| Spain | 3765 | 17 | 3765 | 127 | 3765 | 354 | 3765 | 6 | 3765 | 33 | 3765 | 234 | 3765 | 0 | 3765 | 1066 |
| Finland | 4093 | 6 | 4093 | 82 | 4093 | 154 | 4093 | 10 | 4093 | 18 | 4093 | 77 | 4093 | 0 | 4093 | 311 |
| France | 3954 | 1 | 3954 | 74 | 3954 | 122 | 3954 | 5 | 3954 | 9 | 3954 | 89 | 3954 | 0 | 3954 | 724 |
| UK | 4680 | 23 | 4680 | 181 | 4680 | 556 | 4680 | 8 | 4680 | 46 | 4680 | 263 | 4680 | 0 | 4680 | 1103 |
| Ireland | 4428 | 20 | 4428 | 138 | 4428 | 437 | 4428 | 20 | 4428 | 70 | 4428 | 304 | 4428 | 25 | 4428 | 1670 |
| Netherland | 3734 | 2 | 3734 | 67 | 3734 | 355 | 3734 | 8 | 3734 | 15 | 3734 | 113 | 3734 | 0 | 3734 | 503 |
| Norway | 3374 | 6 | 3374 | 26 | 3374 | 91 | 3374 | 9 | 3374 | 8 | 3374 | 49 | 3374 | 0 | 3374 | 138 |
| Poland | 3619 | 19 | 3619 | 186 | 3619 | 414 | 3619 | 45 | 3619 | 115 | 3619 | 277 | 3619 | 0 | 3619 | 749 |
| Portugal | 4373 | 56 | 4373 | 145 | 4373 | 523 | 4373 | 13 | 4373 | 90 | 4373 | 340 | 4373 | 0 | 4373 | 2150 |
| Russia | 4921 | 44 | 4921 | 267 | 4921 | 940 | 4921 | 71 | 4921 | 276 | 4921 | 701 | 4921 | 0 | 4921 | 946 |
| Sweden | 3774 | 6 | 3774 | 148 | 3774 | 535 | 3774 | 9 | 3774 | 20 | 3774 | 216 | 3774 | 1 | 3774 | 330 |
| Slovenia | 2733 | 10 | 2733 | 57 | 2733 | 386 | 2733 | 18 | 2733 | 32 | 2733 | 208 | 2733 | 5 | 2733 | 636 |
| Slovakia | 3613 | 31 | 3613 | 68 | 3613 | 320 | 3613 | 42 | 3613 | 61 | 3613 | 146 | 3613 | 11 | 3613 | 1340 |

**SM2-Table3. Descriptive statistics of the imputed personal income variable and the standardized version for each year.**

| **Descriptive Statistics** | | | | | | | | | |
| --- | --- | --- | --- | --- | --- | --- | --- | --- | --- |
| TIME | |  |  |  |  | Skewness | | Kurtosis | |
|  |  | N | Mean | Std. Deviation | Variance | Statistic | Std. Error | Statistic | Std. Error |
| 2006 | Personal income | 28021 | 33092.68 | 31982.81 | 1022900428.96 | 1.704 | .015 | 3.427 | .029 |
|  | Standardized personal income | 28021 | 1.034 | .864 | .747 | 3.018 | .015 | 21.941 | .029 |
|  | Valid N (listwise) | 28021 |  |  |  |  |  |  |  |
| 2012 | Personal income | 28640 | 30783.04 | 29259.00 | 856089311.06 | 1.731 | .014 | 4.139 | .029 |
|  | Standardized personal income | 28640 | 1.053 | .643 | .414 | 1.122 | .014 | 1.965 | .029 |
|  | Valid N (listwise) | 28640 |  |  |  |  |  |  |  |

**SM2-Table 4. Partial correlation coefficients for satisfaction, economic and trust parameters used in this study.**

|  |  | **Life satisfaction** | **Social Satisfaction** | **Political satifaction** | **Personal trust** | **Social trust** | **Political trust** | **Personal income** | **Community income** | **Nat’l income** |
| --- | --- | --- | --- | --- | --- | --- | --- | --- | --- | --- |
|  |  | **Post-FC** | | | | | | | | |
| **Pre-FC** | **Life satisfaction** | **1.00** | 0.23 | 0.48 | 0.22 | 0.37 | 0.39 | 0.33 | 0.31 | 0.36 |
|  | **Social Satisfaction** | 0.27 | 1.**00** | 0.24 | 0.21 | 0.32 | 0.24 | 0.21 | 0.19 | 0.17 |
|  | **Political satisfaction** | 0.48 | 0.25 | 1.**00** | 0.06 | 0.47 | 0.74 | 0.42 | 0.46 | 0.52 |
|  | **Personal trust** | 0.19 | 0.18 | 0.06 | **1.00** | 0.08 | 0.05 | 0.02 | 0.00 | -0.03 |
|  | **Social trust** | 0.36 | 0.30 | 0.45 | 0.05 | **1.00** | 0.51 | 0.32 | 0.34 | 0.39 |
|  | **Polt’l. trust** | 0.37 | 0.24 | 0.70 | 0.04 | 0.47 | **1.00** | 0.41 | 0.45 | 0.50 |
|  | **Personal income** | 0.29 | 0.13 | 0.27 | 0.02 | 0.26 | 0.26 | **1.00** | 0.84 | 0.62 |
|  | **Community income** | 0.30 | 0.12 | 0.33 | -0.02 | 0.33 | 0.33 | 0.72 | **1.00** | 0.73 |
|  | **Nat’l income** | 0.35 | 0.16 | 0.42 | -0.05 | 0.37 | 0.41 | 0.50 | 0.68 | **1.00** |

Personal income is measured as yearly household income, community income per thousand is measured aggregated mean of household income for country, region and social class; community income is average of the personal income per region and social class for each country; national income is measured as Ln GDP (PPP) per capita per thousand. Ranges of income: Personal income, -4.45 – 200; Community income, -3-174; National income, 2.4-4.2; Ranges of trust: personal, 1 to 9; social, 0 to 30; political, 0 to 50. Ranges of satisfaction: Social satisfaction, 0.3 to 173.0; Political satisfaction, 0 to 10; Personal life satisfaction, 0 to 10. Significance: * = p < 0.05; ** = p < 0.01; *** = p < 0.001.

**SM2-Table 5. Weighted means or range, and percent change (post-financial crisis relative to pre-) of the primary variables of interest in the study by country and groups of countries based on overall trust. Weighted N= 73,307.**

|  |  |  | **DK** | **NO** | **FI** | **SE** | **CH** | **NL** | **GB** | **DE** | **BE** | **IE** | **ES** | **FR** | **SI** | **CY** | **SK** | **RU** | **PT** | **PL** | **BG** |
| --- | --- | --- | --- | --- | --- | --- | --- | --- | --- | --- | --- | --- | --- | --- | --- | --- | --- | --- | --- | --- | --- |
| **Pre-FC** | **LS** | X̅ | 8.46 | 7.75 | 7.97 | 7.82 | 8.1 | 7.58 | 7.21 | 6.78 | 7.33 | 7.51 | 7.45 | 6.37 | 6.97 | 7.44 | 6.08 | 5.31 | 5.6 | 6.68 | 4.72 |
|  | **Social Satisfaction** | X̅ | 4.7 | 4.88 | 4.47 | 4.69 | 4.68 | 4.36 | 4.05 | 4.43 | 4.31 | 4.47 | 4.54 | 4.29 | 4.5 | 4.64 | 4.17 | 4.01 | 4.54 | 4.25 | 4.12 |
|  | **Polt'l satisfaction** | X̅ | 6.87 | 6.09 | 6.93 | 5.79 | 6.48 | 5.92 | 4.97 | 4.44 | 5.95 | 5.66 | 5.45 | 4.71 | 4.89 | 6.26 | 4.82 | 3.77 | 3.87 | 4.07 | 2.87 |
|  | **Personal income** | X̅ | 46.57 | 61.32 | 36.01 | 39.65 | 62.56 | 38.12 | 49.86 | 31.48 | 34.2 | 50.73 | 26.91 | 32.07 | 17.75 | 29.48 | 11.15 | 5.07 | 17.12 | 8.99 | 3.21 |
|  | **Community income** | R | 57.74 | 109.96 | 51.23 | 75.22 | 158.25 | 172.61 | 130.05 | 85.78 | 62.84 | 76.92 | 170.98 | 136.06 | 59.61 | 118.49 | 35.81 | 11.96 | 97.68 | 79.65 | 11.54 |
|  | **Nat'l income** | X̅ | 3.63 | 3.99 | 3.54 | 3.63 | 3.82 | 3.72 | 3.54 | 3.54 | 3.58 | 3.82 | 3.43 | 3.5 | 3.25 | 3.42 | 3 | 2.7 | 3.23 | 2.72 | 2.43 |
|  | **Personal trust** | X̅ | 3.96 | 3.69 | 3.9 | 3.92 | 4 | 3.71 | 3.79 | 4.05 | 3.64 | 3.96 | 4.01 | 3.44 | 3.98 | 4.1 | 3.78 | 3.89 | 3.95 | 3.96 | 4.1 |
|  | **Social trust** | X̅ | 20.21 | 19.78 | 19.3 | 18.92 | 17.79 | 17.37 | 16.71 | 15.45 | 15.17 | 17.06 | 14.96 | 14.73 | 13.41 | 13.32 | 13.13 | 12.62 | 12.98 | 12.41 | 10.93 |
|  | **Polt'l trust** | X̅ | 32.55 | 27.79 | 31.07 | 27.38 | 28.75 | 27.64 | 22.13 | 22.97 | 24.32 | 23.64 | 23.16 | 21.29 | 19.74 | 25.81 | 20.33 | 16.1 | 18.07 | 15.6 | 11.65 |
| **Post-FC** | **LS** | X̅ | 8.55 | 8.13 | 8.09 | 7.9 | 8.17 | 7.96 | 7.34 | 7.59 | 7.41 | 6.82 | 6.96 | 6.61 | 7.02 | 6.94 | 6.57 | 5.81 | 5.99 | 7.11 | 4.51 |
|  | **Social Satisfaction** | X̅ | 4.88 | 5.01 | 4.55 | 4.82 | 4.83 | 4.56 | 4.4 | 4.6 | 4.47 | 4.72 | 4.64 | 4.46 | 4.71 | 4.4 | 4.21 | 4.1 | 4.55 | 4.47 | 4.32 |
|  | **Polt'l satisfaction** | X̅ | 6.47 | 6.88 | 6.75 | 6.17 | 7.05 | 5.92 | 5.12 | 5.57 | 6.07 | 4.38 | 3.64 | 4.77 | 3.93 | 4.1 | 4.25 | 3.87 | 3.39 | 4.22 | 2.94 |
|  | **Personal income** | X̅ | 50.99 | 74.35 | 38.82 | 46.98 | 83.94 | 39.79 | 6.23 | 35.47 | 33.18 | 30.68 | 22 | 35.02 | 18.11 | 24.42 | 12.69 | 8.21 | 10.43 | 9.21 | 4.12 |
|  | **Community income** | R | 98.31 | 103.14 | 76.45 | 86.08 | 162.92 | 67.09 | 19.23 | 69.15 | 77.04 | 113.72 | 59.16 | 77.62 | 47.04 | 38.81 | 33.27 | 13.48 | 36.99 | 23.46 | 10.37 |
|  | **Nat'l income** | X̅ | 3.83 | 4.18 | 3.71 | 3.81 | 4.06 | 3.85 | 3.63 | 3.78 | 3.75 | 3.86 | 3.49 | 3.68 | 3.36 | 3.46 | 3.29 | 3.23 | 3.32 | 3.17 | 2.81 |
|  | **Personal trust** | X̅ | 4.04 | 3.81 | 3.97 | 4 | 4.02 | 3.8 | 3.86 | 4.09 | 3.71 | 3.97 | 4.03 | 3.61 | 4.12 | 3.99 | 3.85 | 4.01 | 4.03 | 3.98 | 3.94 |
|  | **Social trust** | X̅ | 20.32 | 19.62 | 19.23 | 18.6 | 17.65 | 17.98 | 16.86 | 16.02 | 15.44 | 16.89 | 15.34 | 15.07 | 14.75 | 11.37 | 12.68 | 14.05 | 12.28 | 12.79 | 10.68 |
|  | **Polt'l trust** | X̅ | 32.11 | 30.8 | 31.03 | 28.71 | 30.13 | 28.25 | 23.84 | 25.31 | 24.56 | 21.52 | 16.72 | 21.71 | 16.12 | 17.66 | 15.75 | 16.19 | 14.99 | 16.17 | 11.22 |
| **Percent change** | **LS** | % | 1.03 | 4.87 | 1.49 | 1.11 | 0.82 | 5.05 | 1.76 | 11.93 | 1.1 | -9.15 | -6.52 | 3.84 | 0.73 | -6.68 | 8.14 | 9.55 | 6.95 | 6.43 | -4.45 |
|  | **Social Satisfaction** | % | 3.85 | 2.69 | 1.66 | 2.79 | 3.19 | 4.58 | 8.67 | 3.9 | 3.6 | 5.64 | 2.19 | 4.09 | 4.61 | -5.27 | 1.11 | 2.26 | 0.08 | 5.39 | 4.94 |
|  | **Polt'l satisfaction** | % | -5.89 | 13.09 | -2.6 | 6.57 | 8.79 | 0.04 | 3 | 25.47 | 2.04 | -22.74 | -33.2 | 1.28 | -19.56 | -34.52 | -11.85 | 2.6 | -12.25 | 3.79 | 2.62 |
|  | **Personal income** | % | 9.47 | 21.26 | 7.8 | 18.49 | 34.17 | 4.37 | -87.5 | 12.68 | -3 | -39.52 | -18.24 | 9.21 | 1.98 | -17.16 | 13.87 | 61.94 | -39.1 | 2.51 | 28.13 |
|  | **Community income** | % | 70.25 | -6.2 | 49.22 | 14.44 | 2.95 | -61.13 | -85.21 | -19.39 | 22.6 | 47.85 | -65.4 | -42.96 | -21.08 | -67.24 | -7.11 | 12.74 | -62.14 | -70.55 | -10.18 |
|  | **Nat'l income** | % | 5.57 | 4.81 | 4.78 | 4.89 | 6.18 | 3.54 | 2.62 | 6.69 | 4.6 | 1.03 | 1.81 | 5.09 | 3.46 | 1.27 | 9.94 | 19.53 | 2.73 | 16.67 | 15.65 |
|  | **Personal trust** | % | 1.98 | 3.18 | 1.82 | 2.06 | 0.51 | 2.46 | 1.72 | 0.91 | 2.01 | 0.47 | 0.48 | 4.81 | 3.66 | -2.58 | 1.88 | 3.07 | 2.17 | 0.51 | -3.82 |
|  | **Social trust** | % | 0.53 | -0.79 | -0.36 | -1.69 | -0.76 | 3.53 | 0.88 | 3.64 | 1.75 | -1.04 | 2.53 | 2.36 | 10.02 | -14.65 | -3.42 | 11.26 | -5.32 | 3.05 | -2.36 |
|  | **Polt'l trust** | % | -1.32 | 10.86 | -0.15 | 4.87 | 4.81 | 2.18 | 7.74 | 10.19 | 1 | -8.98 | -27.8 | 1.94 | -18.34 | -31.59 | -22.53 | 0.56 | -17.01 | 3.7 | -3.7 |

Personal income is measured as yearly household income, community income per thousand is measured aggregated mean of household income for country, region and social class; national income is measured as Ln GDP (PPP) per capita per thousand. Ranges of income: Personal income, -4.45 – 200; community income, -3-174; National income, 2.4-4.2; Ranges of trust: personal, 1 to 9; social, 0 to 30; political, 0 to 50. Ranges of satisfaction: Social satisfaction, 0.3 to 173.0; political satisfaction, 0 to 10; personal life satisfaction, 0 to 10. Levels in multilevel: country, region and social class.
